# Recovery of Bioactive Constituents from Olive Leaf Pruning Waste of Five Different Cultivars: A Comparison of Green Extraction Techniques to Maximize Health Benefits

**DOI:** 10.3390/foods14020297

**Published:** 2025-01-17

**Authors:** Hamid Mushtaq, Simona Piccolella, Jose A. Mendiola, Lidia Montero, Elena Ibáñez, Severina Pacifico

**Affiliations:** 1 Department of Environmental, Biological and Pharmaceutical Sciences and Technologies, University of Campania “Luigi Vanvitelli”, Via Vivaldi 43, 81100 Caserta, Italy; hamid.mushtaq@unicampania.it (H.M.); severina.pacifico@unicampania.it (S.P.); 2 Foodomics Laboratory, Institute of Food Science Research CIAL (CSIC-UAM), Nicolás Cabrera 9, 28049 Madrid, Spain; j.mendiola@csic.es (J.A.M.); lidia.montero@csic.es (L.M.); elena.ibanez@csic.es (E.I.)

**Keywords:** *Olea europaea* L., waste valorization, UHPLC-HR MS/MS analysis, UAM, SFE, PLE, bioactive compounds

## Abstract

Sustainable agro-waste revaluation is critical to enhance the profitability and environmental footprint of the olive oil industry. Herein, the valorization of olive leaf pruning waste from five cultivars (‘Caiazzana’, ‘Carolea’, ‘Itrana’, ‘Leccino’, and ‘Frantoio’) employed green extraction methods to recover compounds with potential health benefits. Sequential ultrasound-assisted maceration (UAM) in *n*-hexane and ethanol was compared with a compressed fluid extraction strategy consisting of supercritical fluid extraction (SFE) and pressurized liquid extraction (PLE) for their efficiency in recovering distinct classes of bioactives. Chemical profiling by UHPLC-HR-MS/MS (ultra-high-performance liquid chromatography high-resolution tandem mass spectrometry) and GC-MS (gas chromatography mass spectrometry) showed that UAM-EtOH effectively extracted polyphenols (especially luteolin derivatives) and triterpenes (notably maslinic acid), while PLE yielded the highest amount of secoiridoids (e.g., secologanoside). PLE extracts showed better antiradical activities, putatively due to a higher content of flavonoids, secoiridoids, and HCA derivatives than UAM-EtOH ones, as these latter also contained 20–40% (cultivar-dependent) of triterpenes. SFE extracts with a higher concentration of fatty acids and triterpenes showed moderate antioxidant activities but very high AChE inhibition. This study highlights the importance of selecting appropriate extraction methodologies based on the target bioactive compounds and underscores the potential of olive leaf extracts for sustainable bio-products.

## 1. Introduction

The olive oil industry generates significant amounts of solid, liquid, and solid–liquid waste, mainly from by-products, such as wet pomace, olive leaves, and wastewater [1], which adversely affect the environment, resulting in major concerns like soil and water deterioration and atmospheric pollution [2,3,4]. However, these wastes present interesting opportunities for valorization through innovative practices promoting the principles of sustainability and circular economy [5]. Olive leaves represent a significant by-product, as farmers usually perform the so-called “pruning”, in which they mainly remove thin branches and leaves to improve tree regeneration [6]. The annual amount of pruning waste in Italy can reach 2.6 × 10^9^ kg of dry matter [7]. Indeed, leaves from olive tree pruning are considered residue, having no industrial applications, and are usually burnt or left in the fields for fertilization purposes. Both practices have been associated with severe environmental risks [8], considering that it is estimated that leaves comprise 25% of the dry weight of the entire pruning leftovers and that the expected annual yield of olive leaves in Italy alone is approximately 750,000 tons [9].

In recent years, there has been a growing interest in developing sustainable strategies that not only minimize waste generation but also encourage its valorization, in order to adopt environmental regulations and enhance the profitability of the olive sector. In light of the above, and also considering that the leaves have been recognized as useful reservoirs of bioactive compounds (e.g., phenolics, flavonoids, and triterpenoids) with huge potential health benefits, the reuse of this waste needs to be further promoted. In particular, the possibility of its embedment in a foodstuff with “fortification” purposes could represent a valuable strategy to obtain and successfully exploit innovative functional products.

In this context, the extraction technique plays a pivotal role in recovering these compounds in a wide range of sectors, such as pharmaceutical, cosmeceutical, and food, where they are valued for their therapeutic, nutritional, and functional properties [10,11,12]. However, conventional extraction methods (e.g., maceration, Soxhlet) often make use of large amounts of organic solvents, or high temperatures, and prolonged processing times, which pose environmental hazards as well as leading to the degradation of sensitive compounds or undesirable chemical artifacts. On the contrary, the development of green extraction methods has emerged as a key strategy to improve sustainability while preserving the integrity and efficacy of extracted bioactives. Among the most promising green extraction techniques, ultrasound-assisted maceration (UAM), supercritical fluid extraction (SFE), pressurized liquid extraction (PLE), and microwave-assisted extraction (MAE) have been exploited in recent years [13,14,15]. Each technique shows some benefits and limitations which must be carefully considered in the experimental design. With regard to the ones used herein, UAM utilizes high-frequency sound waves (typically between 20 kHz and 100 kHz) to create cavitation bubbles in the solvent which subsequently collapse and destroy plant cell walls, enhancing the release of bioactives. The main advantages of UAM include reduced extraction time, lower solvent consumption, and the ability to operate at lower temperatures, and, last but not least, UAM can be easily scaled up for industrial applications. It is also compatible with a wide variety of solvents, including green solvents such as water, ethanol, and their mixtures [16,17]. SFE, mostly using supercritical carbon dioxide (scCO_2_) as a solvent, is a popular green technique in the food sector. At supercritical conditions, CO_2_ exhibits both gas-like and liquid-like properties. Thus, it can penetrate the plant matrix like a gas, while dissolving bioactive compounds like a liquid. CO_2_ is non-toxic, non-flammable, and inexpensive, making it an environmentally friendly option. It is especially effective for the extraction of non-polar bioactives, such as essential oils, fatty acids, and terpenes [18]. The primary advantage of SFE is its selectivity and tunability, since, by adjusting temperature and pressure, the solvent strength of supercritical CO_2_ can be fine-tuned to target specific compounds. Its main limitations include the high initial cost of the equipment and the challenge of extracting polar compounds, often requiring co-solvents such as ethanol to improve the efficiency [19]. Finally, PLE uses solvents at elevated temperatures (above the boiling point of the solvent and below the critical point) and pressures enough to keep the solvent in a liquid state. As a result, the process is faster and more efficient in extracting a wide variety of compounds by modulating solvent polarity and operating conditions [20].

The aim of this work is to evaluate and compare the efficiency of two green extraction strategies for the recovery of bioactive compounds from olive leaf pruning waste of five different cultivars collected from the same field and at the same harvesting time. Thus, on the one hand, we employed UAM, and, on the other hand, a sequential strategy engaging SFE followed by PLE was applied to recover first non-polar compounds, and then polar ones. A comprehensive analysis of the extracts, both qualitatively and quantitatively, was achieved by advanced chemical profiling tools (UHPLC-HR MS/MS and GC-MS). Indeed, the applied approach highlights the importance of selecting the most appropriate extraction methodology, based on the bioactives of interest, taking into account that each strategy is responsible for a selective enrichment in certain classes of metabolites. Finally, in order to propose a preliminary perspective in the application to functional food and nutraceutical development, the antiradical and anti-acetylcholinesterase activity were evaluated.

## 2. Materials and Methods

### 2.1. Chemicals and Reagents

*n*-Hexane and ethanol (analytical grade) and formic acid (mass spectrometry grade) were purchased from VWR International (Milan, Italy), whereas water and acetonitrile (mass spectrometry grade) were from Romil Ltd. (Cambridge, UK). CO_2_ used in SFE was purchased from Carburos Metálicos, Air Products Group (Madrid, Spain).

Reagents for antiradical assays were obtained from Merck KGaA (Darmstadt, Germany). Reagents for anti-acetylcholinesterase activity were from Sigma-Aldrich (Madrid, Spain).

### 2.2. Sampling

*O. europaea* L. leaves from five different cultivars (‘Caiazzana’, ‘Carolea’, ‘Itrana’, ‘Leccino’, ‘Frantoio’) were harvested in a commercial orchard owned by the NoveTerre company, located in Caiazzo (Caserta, Italy; coordinates: 41°19′ N, 14°36′ E; Altitude: 200 m a.s.l.) in a typical olive-growing area of southern Italy. In particular, the leaves were randomly collected on 6 April 2022 from the four sides of five olive trees at BBCH stage 19, as described by Sanz-Cortés et al. [21]. Immediately after harvesting, the plant material was transferred to the Food Chemistry Lab, University of the Campania “Luigi Vanvitelli”. A cryo-drying procedure was applied by the FTS-System Flex-dry^TM^ lyophilizer (SP Scientific, Stone Ridge, NY, USA) for 48 h. The water loss was estimated to be equal to 42–47%. All the matrices were powdered and stored at room temperature until further extraction.

### 2.3. Extraction Methods

#### 2.3.1. Ultrasound-Assisted Maceration (UAM)

Dried leaves (1 g) of each cultivar were extracted through ultrasound-assisted maceration (UAM) (Branson Ultrasonics^TM^ Bransonic^TM^ M3800-E, Danbury, CT, USA) sequentially, using first *n*-hexane and then ethanol. In both cases, three sonication cycles (30 min each, frequency of 40 kHz) were performed with a matrix:solvent ratio equal to 1:20 (*w*/*v*). Finally, the obtained extracts were filtered and dried by a rotary evaporator (Heidolph Hei-VAP Advantage, Schwabach, Germany). The dried extracts were stored at 4 °C in the dark until chemical analyses.

#### 2.3.2. Supercritical Fluid Extraction (SFE)

Dried leaves (50 g) of each cultivar were mixed with glass beads (5 mm size; 2:3 ratio) and extracted in a customized speed helix supercritical fluid extractor (Applied Separations, Allentown, PA, USA) using neat as the extraction solvent, as previously described [22]. The experimental parameters were as follows: pressure of 33 MPa, temperature of 60 °C, and CO_2_ gas flow rate of 9 L/min. Extraction kinetics were monitored for a total time of 200 min, collecting extract samples every 20 min (cumulative extraction yield (%) data are shown in Figure S1). Dry extracts were stored at 4 °C in the dark until chemical analyses.

#### 2.3.3. Pressurized Liquid Extraction (PLE)

The dry residues of each cultivar obtained after SFE extract (1 g) were mixed with glass beads (2 mm size; 1:2 ratio) and extracted by PLE using the Dionex ASE 200 accelerated solvent extractor (Thermo Scientific, Waltham, MA, USA). EtOH (80%) was the extracting solvent at 10.3 MPa and 60 °C [23]. Four cycles were performed within a total time of 60 min. The dried extracts were stored at 4 °C in the dark until chemical analyses.

### 2.4. Chemical Characterization

#### 2.4.1. Ultraviolet-Visible (UV-Vis) Spectroscopy and HPLC-UV-DAD Investigation for Pigment Analysis

The extracts obtained using the different extraction techniques were reconstituted in the most suitable solvent (0.1 mg/mL in *n*-hexane or EtOH, based on the polarity) and underwent UV-Vis spectrophotometric measurement vs. a blank in the range of 200–800 nm by a Cary 100 instrument (Agilent, Milano, Italy). Three independent replicates were carried out. The pigment content (expressed in ppm) was calculated, according to the formulas for total chlorophylls (3) and for total carotenoids (4) from [24]:

Chlorophyll a = 9.93 × A663 − 0.78 × A640(1)

Chlorophyll b = 17.60 × A640 − 2.81 × A663(2)

Chlorophyll a + b = 7.12 × A663 + 16.80 × A640(3)

Total carotene = (1000 × A470 − 0.52 × Chl a − 7.25 × Chl b)/226(4)

**Figure 1:**
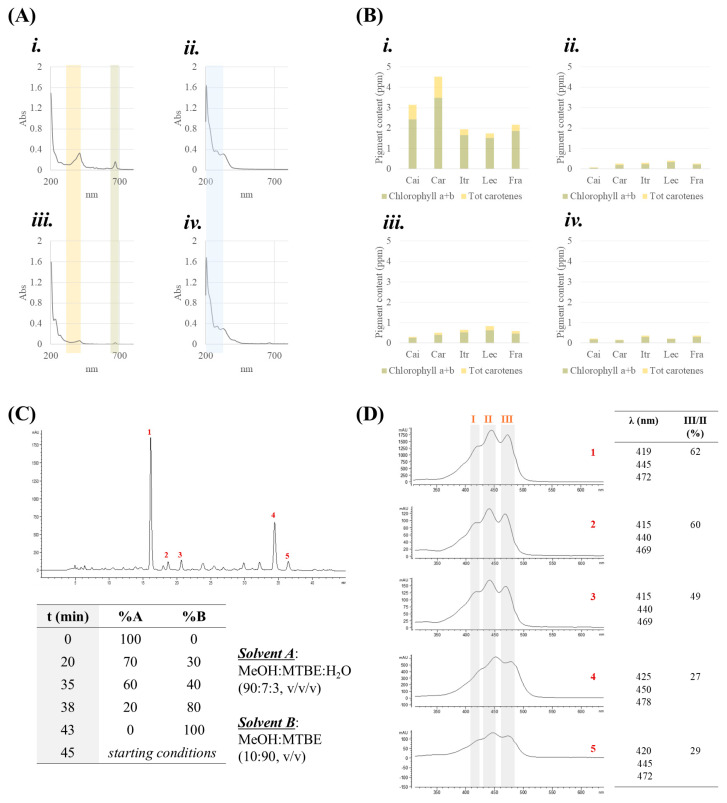
(**A**) Representative UV-Vis spectra and (**B**) pigment content of (**i.**) UAM *n*-hexane, (**ii.**) UAM EtOH, (**iii.**) SFE, and (**iv.**) PLE extracts. (**C**) LC-UV-DAD chromatogram of carotenoids, and (**D**) their UV spectra.

Carotenoids were also investigated by HPLC-UV-DAD. For this purpose, an Agilent 1100 Series HPLC (Agilent, Santa Clara, CA, USA) was used, equipped with a diode array detector (DAD). The separation was achieved on Carotenoid C30 Column (250 × 4.6 mm i.d., 5.0 μm particle size, YMC America, Devens, MA, USA), with a gradient of MeOH:MTBE:H_2_O (90:7:3, *v*/*v*/*v*) (solvent A) and MeOH:MTBE (1:9, *v*/*v*) (solvent B). The detailed linear gradient is shown in Figure 1. The flow rate was set atot 0.8 mL min^−1^, and the injection volume was 10 μL. The separation was acquired at 450 nm.

#### 2.4.2. HPLC-HRMS and MS/MS Analysis

UHPLC-HRMS untargeted analysis was carried out to unravel the chemical composition of UAM *n*-hexane and ethanolic extracts. A targeted MS approach was then used to characterize PLE and SFE extracts. For this purpose, a NEXERA UHPLC system (Shimadzu, Tokyo, Japan) equipped with a Luna^®^ Omega C18 column (50 × 2.1 mm i.d., 1.6 µm particle size) (Phenomenex, Torrance, CA, USA) was coupled with the AB SCIEX Triple TOF^®^ 4600 (AB Sciex, Concord, ON, Canada). The elution gradient, made of water (A) and acetonitrile (B), both containing 0.1% formic acid, took into consideration the chemical complexity of the samples and was optimized to achieve the best peak resolution. Detailed information on the gradients applied is reported in Figure S2. HRMS and MS/MS spectra were recorded in negative electrospray ionization (ESI) mode, following automated mass calibration in all scan functions via the APCI probe. The untargeted approach combined TOF-MS and MS/MS with information dependent acquisition (IDA), consisting of a full scan TOF survey (accumulation time 250 ms, 200–1200 Da) and eight IDA MS/MS scans (accumulation time 100 ms, 95–1000 Da). The ESI source parameters were the following: curtain gas (CUR) of 35 psi, nebulizer gas (GS 1) of 60 psi, heated gas (GS 2) of 60 psi, and ion spray voltage (ISVF) of 4.5 kV. Moreover, other parameters were optimized based on the extract type: an interface heater temperature of 600 °C (or 500 °C), a declustering potential of a −70 V (or −80 V), and a collision energy of −40 ± 5 V. The instrument was controlled by the Analyst^®^ TF 1.7 software, while data processing was carried out using the PeakView^®^ software version 2.2.

#### 2.4.3. GC-MS Analysis

SFE extracts, previously dissolved in ethanol and filtered on Sartorius NY 0.45 μm filters, were also analyzed by using the QP2010 Plus GC-MS system (Shimadzu, Tokyo, Japan) equipped with a Zebron ZB5-MS capillary column (30 m × 250 μm i. D. × 0.25 μm) (Phenomenex, Torrance, CA, USA). Helium was used as a carrier gas at a constant flow rate of 0.8 mL min^−1^. The injection volume was 10 μL in split mode (1:10), setting the injector temperature to 250 °C. The initial oven temperature was set to 45 °C, then it increased to 200 °C in 15 min before reaching 300 °C at a rate of 5 °C/min, and then it was held for 15 min.

The MS detection was achieved by operating in full-scan acquisition mode at an *m*/*z* scan range of 40–500 Da, with the interface and source temperature set to 335 °C and 250 °C, respectively.

Data were processed using the GC-MS solution (ver. 2.71, Shimadzu) software. The compounds were tentatively identified by comparing the recorded MS spectra with those in available mass spectral databases (Wiley and NIST).

### 2.5. Antiradical Assessment

The antiradical capacity of the extracts from UAM, SFE, and PLE was evaluated by DPPH and ABTS methods [25], employing the following final doses: 4, 20, 40, 100, and 200 µg/mL. In both tests, 6-hydroxy-2,5,7,8-tetramethylchroman-2-carboxylic acid, hereafter mentioned by its commercial name Trolox^®^, was used as the positive standard at 2, 4, 8, 16, and 32 µM final concentration levels. The absorbance was read at 520 and 720 nm, respectively, by a Wallac Victor^3^ spectrophotometer (PerkinElmer, Waltham, MA, USA) against a blank sample, containing only the radical probe, arranged in parallel. The results were expressed in terms of ID_50_ values based on the percentage decrease of the initial radical probe absorption due to the co-presence of the samples. TEAC (Trolox^®^ equivalent antioxidant capacity) values were also calculated as ID_50_Trolox/ID_50_sample [26].

### 2.6. Anti-Acetylcholinesterase (AChE) Activity

The anticholinergic activity of the PLE and SFE extracts was assessed against the acetylcholinesterase (AChE) enzyme, using Ellman’s method modified by a fluorescent enzyme kinetics study with ABD-F as the fluorescent probe. The experimental setting has been previously detailed [22]. Galantamine (0.42–4.2 µg/mL final doses) was the positive control, whereas the negative control samples did not contain the inhibitors. PLE extracts were tested in the range of 66.7–666.7 µg/mL, whereas SFE extracts in the range of 16.7–166.7 µg/mL (seven dose levels each).

ID_50_ values were calculated, representing the concentration (expressed as μg/mL) of galantamine or extracts that produced 50% of cholinergic enzyme inhibition capacity compared with the control (without inhibitors). Therefore, lower ID_50_ values means higher anti-acetylcholinesterase activity.

### 2.7. Data Processing and Statistical Analysis

Results are expressed as mean values ± standard deviation deriving from two independent experiments performed in triplicates. Means were compared using a one-way ANOVA plus a Dunnett’s multiple comparison test. A *p*-value of < 0.05 was considered to indicate a statistically significant result. The GraphPad Prism 8 software (Graphpad Software. La Jolla, CA, USA) was used to perform heat maps of sample chemical composition. Additionally, the OriginPro 2015 software (OriginLab Corp., Northampton, MA, USA) was used for multivariate analysis methods (Hierarchical Cluster Analysis and Principal Component Analysis (PCA)).

## 3. Results and Discussion

The extraction of bioactive compounds from olive leaves is essential for leveraging their potential health benefits and promoting sustainable practices within the olive industry [27]. Herein, ultrasound-assisted maceration (UAM) was initially employed. This extraction method is recognized for its simplicity, cost-effectiveness, and ability to enhance the yield of bioactive constituents. Notably, a biorefinery strategy was implemented within the UAM using *n*-hexane to obtain a first fraction rich in non-polar compounds, followed by ethanol to extract a second fraction containing more polar bioactive compounds. This dual-fraction approach allowed us to maximize the recovery of a diverse array of phytochemicals, including (poly)phenols, and triterpenes, which are known for their antioxidant and anti-inflammatory properties. While the yields obtained by UAM were promising (Table S1), the integration of supercritical fluid extraction (SFE) and pressurized liquid extraction (PLE) as part of a comprehensive greener biorefinery strategy was also explored. The rationale for this transition was two-fold: first, the CO_2_ used in SFE can replace *n*-hexane; second, PLE enhances the extraction of polar compounds, including phenolic acids and flavonoids, by operating under elevated temperatures and pressures, thereby improving extraction efficiency and reducing processing times. By combining SFE and PLE with the initial UAM approach, a comprehensive approach was optimized to maximize the recovery of valuable phytochemicals from olive leaves, also demonstrating that each strategy is responsible for selective enrichment in certain classes of metabolites.

### 3.1. Spectrophotometric and HPLC-DAD Analysis of Carotenoids and Chlorophylls

All the samples obtained, regardless of the extraction technique, underwent UV-Vis spectrophotometric analysis, which gave a first picture of the ability of each technique to extract carotenoids and chlorophylls. From a qualitative point of view, the five cultivars showed almost superimposable spectra regarding absorption bands, differing only slightly in intensity. In Figure 1A, representative UV-Vis spectra are reported for each extraction technique. It is prompt to observe that UAM *n*-hexane and SFE samples were comparable, as well as the UAM EtOH and PLE ones, suggesting the presence of a similar chemical composition in the main compound classes. Indeed, the absorption bands in the range of 270–290 and 330–340 nm were in line with the presence of phenolic secoiridoids and flavonoids in the most polar extracts. On the other hand, the spectra of apolar samples were dominated by two main bands, characteristic of carotenoids and chlorophylls at 412 and 667 nm, respectively. There are two main forms of chlorophyll, *a* and *b*, differing for chemical structure and biological roles. Chlorophyll *a* is the primary pigment involved in photosynthesis, while chlorophyll *b* acts as an accessory pigment, enhancing light absorption and energy conversion [28]. The amount of chlorophyll strongly influences the color of olive leaves; it has been reported that its content varies across different cultivars and also with leaf age, which also affects the retention of carotenoids [29]. Beyond their role in photosynthesis, these pigments are appreciated as food additives and colorants in the food industry, but also for their functional properties. Olive leaves, rich in both carotenoids and chlorophyll, have a great potential for food functionalization. One promising application is the incorporation of pigment- rich olive leaves into olive oil, an approach which has been shown to enhance the oil nutritional quality and which could open up new market opportunities in the food industry [30]. In this work, it was found that the amount of both pigments was dependent on the applied extraction technique, beyond the cultivar (Figure 1B). Indeed, UAM with *n*-hexane was able to provide their highest content when compared to SFE, with ‘Carolea’ as the richest cv., followed by ‘Caiazzana’. Considering that both pigments are fat-soluble, it is not surprising that the most polar extracts (UAM EtOH, and PLE), obtained from plant matrices previously defatted, showed only a minor content. To get further insights into the carotenoid composition, an HPLC-DAD analysis was developed using a C30 column (Figure 1C). As an example, Figure 1C shows the carotenoid profile of the SFE from the ‘Carolea’ cultivar. Among the separated peaks, five compounds were detected as carotenoids by the evaluation of their UV-Vis spectra, which showed their typical light absorption. Indeed, a characteristic three-absorbance maximum spectrum in the range of 400–500 nm was detected. These maximums are correlated with their highly conjugated π system in the chemical structure. Some peculiar features are commonly used to identify them with good accuracy. Briefly, besides the wavelengths of each peak (I, II, and III; Figure 1D), which could differ depending on the carotenoid structure, the intensity ratio (%) between peaks III and II could also offer great discrimination opportunities [31]. Thus, the comparison with literature data allowed us to propose the presence of lutein and β-carotene isomers [32,33]. In particular, spectra 1–3 (Figure 1D) were attributed to (*all-E*)-lutein, based on λ_max_ values at 419, 445, and 472, and the % III/II value equaled 62. Moreover, the ~4 nm shift observed for peaks 2 and 3, together with the low intensity of the so-called *cis* peak in the near UV region (at 330 nm) [34], was in accordance with the presence of (9*Z*)- or (9′*Z*)-lutein geometrical isomers [33]. In fact, this additional absorption band was found to increase as the *cis* double bond was located in the center of the molecule. Peaks 4 and 5 were, putatively, (*all-E*)-β-carotene and its (9*Z*)-isomer, following similar observations. Indeed, lutein and β-carotene were also described as carotenoid constituents of olive leaves from cultivars Giarraffa and Olivastra Seggianese [35].

### 3.2. UHPLC-HRMS Investigation of UAM Extracts

#### 3.2.1. Chemical Characterization

The metabolite chemical profile of the olive leaves from the five cultivars was carried out by an untargeted UHPLC-HRMS approach applied to two extracts with different polarities, obtained by sequential UAM procedures, i.e., *n*-hexane and ethanol. From a qualitative point of view, regardless of the cultivar, non-polar extracts were constituted mainly by free fatty acids (FFAs). Moreover, one triterpene compound, tentatively identified as acetyl oleanolic acid, was also tentatively detected (Figure S3). This latter, showing a deprotonated molecular ion at *m*/*z* 497.3639, lost the acetyl group as acetic acid (−60 Da), generating the fragment ion at *m*/*z* 437.1416 (−2.1 ppm error). Very few traces of the other ursane and/or oleanane-type triterpene were not considered, as they were detected in a very low intensity. The identification of FFAs followed previous investigations of our research group [36]. UHPLC-HRMS data are summarized in Table 1.

The relative abundance of each detected metabolite, calculated as % based on the peak areas from extracted ion chromatograms (XICs), allowed us to highlight peculiar differences in each fatty acid within the same extract and among the five cultivars. All the samples mainly accounted for polyunsaturated fatty acids (PUFAs), which were especially high in the ‘Caiazzana’ cultivar (82%; Figure S4). Among them, α-linolenic acid was the main constituent. The ‘Itrana’ cultivar was the sample with the highest content of this PUFA (44.5%), whereas ‘Carolea’ showed the lowest content (25.4%). Instead, the ‘Caiazzana’, ‘Leccino’, and ‘Frantoio’ samples had comparable amounts, in the range of 35.2–37.7%.

UAM-ethanol extracts obtained after the *n*-hexane step were also analyzed to investigate the richness in polar bioactives, mainly composed by phenolic compounds and triterpenes, in addition to secoiridoid constituents. Sixty-three metabolites were revealed and grouped into seven classes, based on their tentative chemical identification (simple sugars, glycosidic aroma precursors, hydroxycinnamic acid derivatives, secoiridoids, flavonoids, and pentacyclic triterpenes). For their identification, literature data for comparison were taken into account when available. In particular, a recent paper by our research group on ‘Caiazzana’ leaf polar constituents [37] was pivotal herein for an initial dereplication study. Considering that the collection year, season, and site, as well as the genotypes and the tree phenological stage, were different, it is reasonable to assume that slight changes in the qualitative/quantitative profile may occur. In Table 1, the whole UHPLC-HRMS untargeted profile is reported. However, only the identification of compounds that differed from those previously reported [37] will be further deepened, whereas, for all the others (labeled in Table 1 with an asterisk), refer to the previous work.

In the first region of the chromatogram, a dihexose (at *m*/*z* 341.1089) was found together with a glycosyl alditol at *m*/*z* 343.1249 (Figure S5, panels A and B). A further glycosidic aroma precursor was undisclosed, namely benzyl primeveroside (C_18_H_26_O_10_), previously found in olive by-product extracts [38]. Apart from verbascoside, other six hydroxycinnamic acid derivatives were detected (Figure S6), among which campneoside II (β-hydroxyverbascoside), previously found in olive drupes [39], and osmanthuside B were tentatively identified in this sample. This latter differs from verbascoside for the presence of a *p*-coumaroyl (instead of caffeoyl) moiety and has been proposed as the precursor of verbascoside [40]. Moreover, hexosyl derivatives of caffeic (*m*/*z* 341.0882) and ferulic acid (*m*/*z* 355.1031), as well as the hexytolylhexoside derivative of ferulic acid (*m*/*z* 519.1725), were detected at 1.332, 3.292, and 3.000 min, respectively. Their fragmentation consisted of the loss of the glycosidic moieties, giving clear information on the aglycone identity. Secoiridoids other than oleuropein, its hexoside and hydroxy derivatives, were detected as well (Figure S7). In particular, oleoside and its isomer secologanoside (C_16_H_22_O_11_) and three hexosides of elenolic acid (C_17_H_24_O_11_) were observed [41]. Recently, a systematic mass spectrometry characterization was carried out, highlighting isoforms of oleuropein, their demethylated metabolites, and elenolic acid glucosides from fruits and leaves of the ‘Coratina’ cultivar, providing useful information on the structural discrimination [42]. Finally, according to its fragmentation pattern, the compound at *m*/*z* 535.1477 (C_25_H_28_O_13_), detected at 9.077 min, was tentatively identified as *p*-coumaroyl secologanic acid [43].

It is worth noting that, despite secoiridoids having attracted broad interest as the main bioactive constituents of olive oils and also having been detected in wastes and by-products from the olive oil production chain, in the UAM extracts herein investigated, flavonoids and pentacyclic triterpene acids were found in considerable amounts. Flavonoids were mostly represented by flavone (i.e., apigenin, luteolin, and diosmetin) glycosides [44,45], apart from quercetin, and eriodictyol skeletons. As discussed above, the chemical characterization will be limited to those compounds not labeled with a star key in Table 1. The apigenin derivative at 3.745 min (*m*/*z* 593.1520; C_27_H_30_O_15_) was the only *C*-glycoside detected, as suggested by its fragmentation pattern, which showed neutral losses corresponding to the sugar cross-ring cleavages (−90, −120 Da; Figure S8, panel A) instead of a dehydrated sugar moiety (−162 Da), as occurred in the *O*-hexoside eluting at 7.571 (*m*/*z* 431.1000; C_21_H_20_O_10_). The aglycone ion intensity of this latter was in accordance with a 7-*O* substitution (Figure S8, panel B). The tentative identification of luteolin and diosmetin glycosides followed the same rules, where dehydrated disaccharides (dihexose or deoxyhexosylhexose) were recognized by a one-step neutral loss (−324 Da and −308 Da, respectively) giving the deprotonated aglycone. On the contrary, when the two sugars were linked at two different positions, two independent fragment ions were formed, each one corresponding to the loss of one monosaccharide unit (Figures S9 and S10). Luteolin *p*-coumaroylhexoside was also tentatively identified at 10.646 min, thanks to the accurate mass measurement that gave the molecular formula C_30_H_26_O_13_, according to the presence of acyl moiety instead of deoxyhexose. The flavone base skeleton did not further fragment under the experimental conditions applied. On the contrary, HR-MS/MS spectra of flavonol and flavanone glycosides showed fragment ions attributable to the aglycone B ring at lower *m*/*z* values (e.g., *m*/*z* 271, 179 and 151; Figure S11), thus confirming the structure hypothesis.

Due to their polarity, lower than secoiridoids and flavonoids, pentacyclic triterpenes were eluted in reversed-phase chromatography only when the acetonitrile % in the mobile phase increased rapidly. The olive leaves are recognized as abundant sources of triterpenic acids and pentacyclic triterpenols, which can be found either in their free form or glycosylated [28], with oleanolic acid as the most abundant triterpenic acid compound [46]. It is worth noting that, in the mass spectrometry experimental conditions, the fragmentation of the pentacyclic core is poor. Thus, whenever present, the only detected fragments were referred to hydroxyl groups lost as water molecules, as previously reported [47]. In addition to oleanolic acid, four isomeric dihydroxy derivatives were found. In fact, their MS spectra showed the deprotonated molecular ion at *m*/*z* 487.3431(4), whose molecular formulas (C_30_H_48_O_5_) have two more oxygen atoms than oleanolic acid. A hydroxyl oleanolic acid (e.g., maslinic acid) at *m*/*z* 471.3480 was also detected, together with the hydroxy-oxo-oleanonic acid at *m*/*z* 469.3320. Moreover, ursolic and betulinic acids, sharing the same deprotonated ions at *m*/*z* 455.353 but different retention times, were tentatively identified (Figure S12).

#### 3.2.2. Principal Component Analysis (PCA) of Compound Classes Detected in the UAM EtOH Extracts

**Figure 2:**
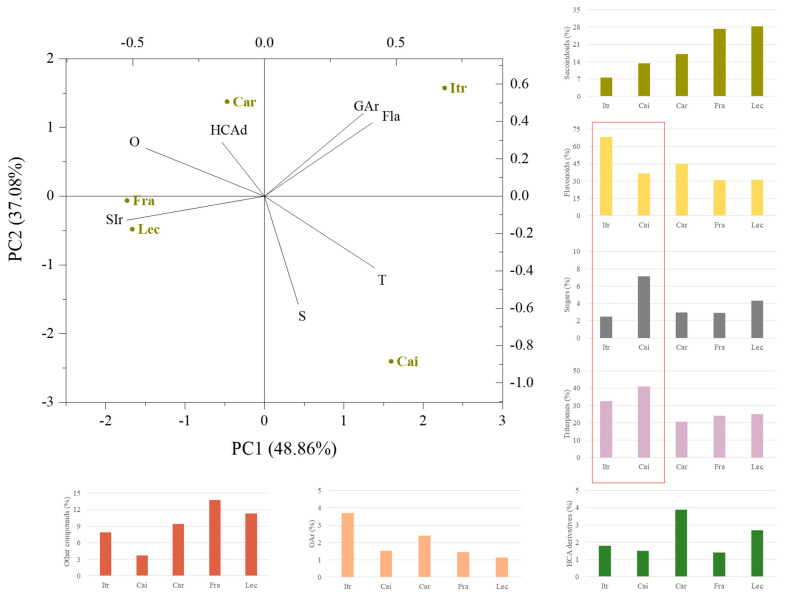
Principal component analysis (PCA) biplot of the data matrix obtained for UAM EtOH extracts from the olive cultivars under investigation (Cai = ‘Caiazzana’, Car = ‘Carolea’, Itr = ‘Itrana’, Lec = ‘Leccino’, Fra = ‘Frantoio’). Data were processed by using the OriginPro 2015 software. Representative variables were plotted also as histograms. Fla = flavonoids; GAr = glycosidic aroma precursors, HCAd = hydroxycinnamic acid derivatives; S = sugars; SIr = secoiridoids; T = triterpene; O = others.

The relative quantitation of the compounds tentatively identified, grouped in their chemical classes, was useful in revealing compositional differences in the five cultivars under study. The results were firstly analyzed by a multivariate statistical approach through principal component analysis (PCA) (Figure 2).

The first two principal components (PC1 and PC2) explained 85.94% of the total variance in the dataset. It clearly shows that, along PC1, the cultivars were mainly distinguished by their content of secoiridoid compounds, which mostly characterized ‘Frantoio’ and ‘Leccino’, whereas ‘Itrana’ was the poorest cultivar. Moreover, following PC2, the sugar content allowed to differentiate thoroughly the ‘Itrana’ and ‘Caiazzana’ samples. The high abundance in ‘Caiazzana’ leaves could explain the reversed trend observed for flavonoids. Indeed, to the best of our expertise, simple carbohydrates in a mixture with polyphenols affect the ESI ionization of these latter ones in negative ion mode, resulting in ion suppression due to a matrix effect. Thus, the sugar removal through fractionation techniques proved to unmask their presence and richness [48]. On the contrary, triterpene compounds seem to be not correlated with this negative effect, likely due to the acidic carboxylic groups that are more susceptible to fast deprotonation. Among the investigated samples, ‘Caiazzana’ leaves were the richest source of these specialized metabolites. Deepening into the flavonoid subclasses, it was found that flavones were the most representative not only considering the high number of their derivatives (23 compounds vs. 3 flavonols and 1 flavanone), but mostly taking into account their relative percentage. In particular, the peculiar richness in luteolin and its glycosides was highlighted by heat map analysis, among which luteolin hexoside II was likely responsible for ‘Caiazzana’ segregation in the hierarchical cluster analysis branch II (Figure S13). Furthermore, the dissimilarity of ‘Itrana’ leaves (subcluster I.b) could be due to apigenin and diosmetin glycosides.

### 3.3. SFE Extracts

As an alternative and greener extraction to UAM using *n*-hexane, the olive leaves from the five cultivars underwent supercritical fluid extraction using scCO_2_, avoiding the use of any organic extraction solvent. Carbon dioxide is generally recognized as safe (GRAS), meaning that products obtained following its use are considered safe for human consumption [49]. ScCO_2_ SFE is particularly effective for extracting non-polar bioactive compounds, making it ideal for targeting compounds such as fatty acids and terpenes from olive leaves. In this work, the extraction yields were monitored every 20 min until 200 min (Figure S1), in order to understand the time-dependent efficacy of this technique in solubilizing the main targeted compounds, finding a sort of compromise between time and yield increase rate. Across all five cultivars, a similar pattern was observed: extraction rates increased significantly during the first 80 min, followed by a plateau where further increases in time and CO_2_ consumption did not enhance yields. This yield plateau suggests that 80 min may be the optimal extraction time, balancing efficiency and resource consumption.

#### 3.3.1. Metabolite Targeted Profiling by UHPLC-HRMS: A Comparison with Non-Polar Hexane UAM

A targeted UHPLC-HRMS approach was developed for the five SFE extracts, based on the chemical constituents previously undisclosed and tentatively identified in UAM extracts. The aim was to compare the two techniques beyond the yields, based on their selective extraction capability. As shown in Table 2, the enrichment in FFAs was comparable among the five cultivars from a qualitative point of view, whereas pentacyclic triterpenes were detected in the SFE samples as minor constituents.

**Figure 3:**
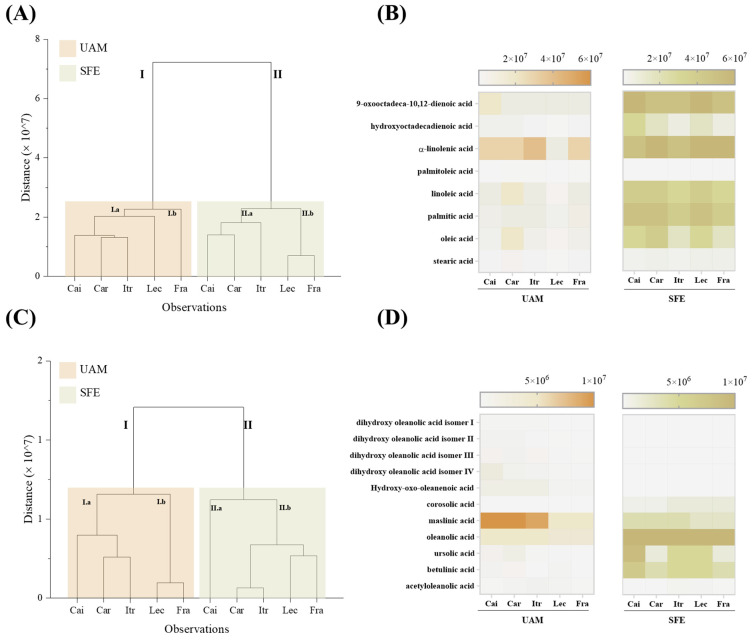
Hierarchical cluster analysis of (**A**) FFAs and (**C**) pentacyclic triterpenes in UAM (*n*-hexane extracts) and SFE extracts from the five cultivars (Cai = ‘Caiazzana’, Car = ‘Carolea’, Itr = ‘Itrana’, Lec = ‘Leccino’, Fra = ‘Frantoio’) under study, and the corresponding heat map analyses (**B**,**D**).

Hierarchical cluster analyses of UAM and SFE extracts were performed, considering FFAs and pentacyclic triterpenes separately (Figure 3). Despite the metabolite class, dendrograms were divided into two main branches (I and II), which correspond to the samples deriving from UAM (brown color) or SFE (light green color). It is the first evidence that the extraction technique is the main responsible variable for different metabolite enrichment. It was particularly evident when FFAs were considered (Figure 3A). The distance between the two branches could be ascribed to a higher content of linoleic, palmitic, and oleic acid, in addition to oxidized 18:2 acids, during SFE (Figure 3B).

Each branch appeared to be further divided into two subclusters (labeled with the letters “a” and “b”), highlighting slight dissimilarity among the five cultivars subjected to the same extraction method. Regarding triterpenes, oleanolic acid was the main C_30_H_48_O_3_ isomer in all the samples, whereas the detected amount of its hydroxyl derivative maslinic acid could explain the dissimilarity of subcluster I.b (Figure 3C), including the ‘Leccino’ and ‘Frantoio’ cultivars. The presence of dihydroxy oleanolic acid isomers, detected in UAM extracts, was negligible in SFE samples. It is worth noting that the ‘Caiazzana’ SFE extract alone constituted the subcluster II.a, likely due to the ursolic acid, whose content was 1.6–4.3-fold higher than that of the other samples. On the contrary, its hydroxylated derivative corosolic acid, which was not previously detected applying UAM, was more abundant in the ‘Itrana’, ‘Leccino’, and ‘Frantoio’ cultivars.

#### 3.3.2. GC-MS Profiling of SFE Extracts: A Comparison with LC-MS

GC-MS is widely recognized for its ability to detect volatile and semi-volatile compounds with high reproducibility [50]. In this study, GC-MS was employed to profile the analytes extracted by SFE from olive leaves, and the results are summarized in Table 3. Compound identification was performed by comparison with the NIST and Wiley MS databases provided with the instrument, based on the spectra similarity score (%). It should be noted that, although the total number of detected compounds by GC-MS was very similar to that from UHPLC-HRMS (16 vs. 14), only three metabolites were detected through both techniques, namely palmitic, linoleic, and linolenic acid (Figure S14) in olive leaf extracts. It is likely ascribed to the peculiar features of both analytical techniques, which make them suitable for detecting, selectively, some compound classes. Thus, taken together, data obtained from both techniques on the same sample can be successfully matched to get complementary information, enlarging the knowledge of its metabolic composition. Among the others, the presence of one fatty aldehyde, three hydrocarbons, and four triterpene alcohols were revealed, in addition to lower amounts of β-sitosterol, α-tocopherol and its isomer (Figure S15), and the sesquiterpene globulol [22].

Their relative amount, expressed as %, is reported in Figure S16. The fatty acids identified were present in significant amounts across the cultivars. They are known for their health-promoting properties, including anti-inflammatory and cardiovascular benefits [32]. Additionally, the presence of squalene and β-sitosterol further underscores the potential of olive leaf extracts for nutraceutical and cosmetic applications, as these compounds are valued for their antioxidant and skin-protective properties [47]. Moreover, GC-MS detected *n*-alkanes such as heneicosane and tetratriacontane, both of which are often found in the plant leaf cuticular waxes. These compounds play a role in plant defense and have also been identified as potential markers for authenticity in olive oil products [51]. In terms of triterpenes, besides two lupeol isomers (Figure S17) [52], erythrodiol and urs-12-en-28-ol were among the key compounds identified, both of which are precursors to oleanolic acid and maslinic acid, the main triterpenic acids of olive leaves. These triterpenes have been extensively studied for their anti-inflammatory, anti-cancer, and anti-microbial properties, suggesting that olive leaf extracts could serve as valuable sources for developing functional ingredients [53]. The untargeted GC-MS analysis provided valuable insights into the volatile and semi-volatile profiles of the olive leaf extracts, highlighting their potential for diverse industrial applications, including functional foods, cosmetics, and pharmaceuticals. Given the simplicity and reproducibility of the method, GC-MS proved to be a robust tool for profiling specific bioactive compounds, especially those involved in fatty acid and triterpene metabolism.

#### 3.3.3. PLE vs. UAM: Metabolite Targeted Profiling by UHPLC-HRMS

**Figure 4:**
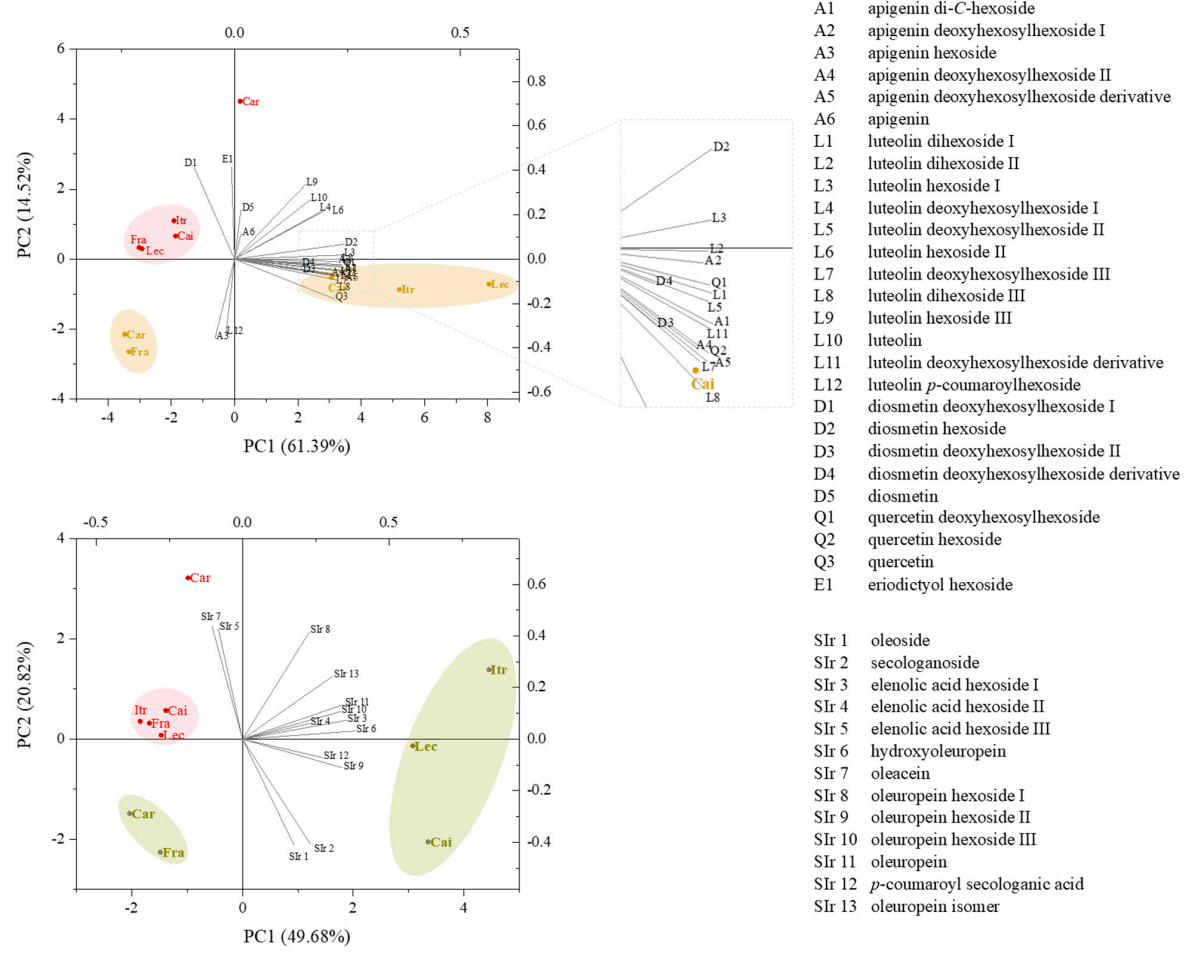
Principal component analysis (PCA) biplot of the data matrix for flavonoids and secoiridoids from olive leaf extracts (Cai = ‘Caiazzana’, Car = ‘Carolea’, Itr = ‘Itrana’, Lec = ‘Leccino’, Fra = ‘Frantoio’). UAM-EtOH extracts are labeled in red, whereas PLE is green/yellow. Data were processed by using the OriginPro 2015 software.

Once the olive leaves were defatted by SFE, they underwent sequential PLE. Thus, it was reasonable to assume that the chemical constituents were comparable to those detected in UAM EtOH extracts. In order to make a fast, reliable, and straightforward comparison between the two extraction techniques, the metabolic complexity therefrom was investigated through a targeted UHPLC-HRMS approach, based on the chemical characterization previously performed (reported in Section 3.2), leading to the identification of 47 compounds (Table 4). A representative total ion current chromatogram (TIC) is reported in Figure S18 (panel A), in which compounds under peaks were labeled with different colors, based on the class to which they belong. PLE extracts were mostly enriched in flavonoids and secoiridoids, accounting for about 80% of the total metabolic content, with ‘Caiazzana’, ‘Itrana’, and ‘Leccino’ showing the richest metabolite variability, also considering hydroxycinnamic acid derivatives (HCAd) (Figure S18, panel B). In the ‘Carolea’ sample, the sugar occurrence was far higher than that in the other cultivars, leading to a ~20% decrease in the specialized metabolites. Among the compounds classified as “other”, it is worth noting that hydroxytyrosol hexoside mainly characterized the ‘Leccino’ and ‘Frantoio’ cultivars (6.3 and 4.6%, respectively), as previously observed in UAM extracts from the same matrices. Triterpene compounds were undetectable, likely due to their exhaustive extraction in the previous SFE step. A deeper investigation was carried out on the main metabolite classes, i.e., flavonoids and secoiridoids. To this purpose, a multivariate statistical analysis approach was applied, comparing the data *per* cultivar and *per* extraction technique (PLE vs. UAM-EtOH). The results were globally visualized by a PCA biplot (Figure 4). With regard to flavonoids, the first two components were able to explain 75.91% of the total variance in the data set, where the extraction-based grouping in positive and negative half-planes showed a clear separation between UAM and PLE techniques. Moreover, this latter granted a higher differentiation among the five cultivars. ‘Carolea’ and ‘Frantoio’ were distinguishable from the other three samples for the lower content of most luteolin glycosides. Furthermore, they were characterized by higher amounts of luteolin *p*-coumaroylhexoside and apigenin hexoside. On the contrary, except for the ‘Carolea’ sample, when compared to PLE, UAM flavonoids alone could not discriminate among the cultivars, whose scores appeared to be grouped in the upper left quadrant of the biplot. Although the total variance in the secoiridoids dataset was slightly lower (70.50%), the comparison of EtOH-UAM with PLE extracts showed a similar segregation of UAM samples, in which oleacein mainly characterized ‘Carolea’. This metabolite was not detected in the PLE extracts. Among them, ‘Carolea’ and ‘Frantoio’ were, again, located on the negative side of PC1. Oleuropein and its derivatives were the main responsible for this segregation, whereas ‘Itrana’, ‘Leccino’, and ‘Caiazzana’ could be better discriminated along PC2. Indeed, apart from an increase in the content of oleuropein and hydroxyoleuropein (‘Itrana’ > ‘Leccino’ > ‘Caiazzana’), their separation could be also explained thanks to the relative amount of secologanoside and oleoside that followed the opposite trend.

### 3.4. Antiradical and Anti-AChE Activity Assessment

The antioxidant capacity was evaluated using the DPPH and ABTS assays, widely recognized for their ability to assess the radical scavenging potential of bioactive compounds [26]. Trolox, a water-soluble analog of vitamin E, was used as positive control to standardize the antioxidant potential (TEAC values). It should be noted that the response to these assays is strongly dependent on the chemical composition of the tested samples. Thus, UAM *n*-hexane extracts were discarded from the experimental design, being constituted of metabolites that could hardly act as donors of a single electron or hydrogen atom to neutralize the radical probes, due to their chemical structures. Moreover, the SFE extracts displayed a very low antioxidant capacity, likely due to their enrichment in non-polar compounds, such as fatty acids and triterpenes [8]. Fatty acids like linoleic and oleic acid, while beneficial in other contexts (e.g., cardiovascular health), are less effective as direct antioxidants compared to polyphenols. Indeed, although α-tocopherol and its isomer were detected in SFE extracts, their percentage in the mixture (2.3% or lower) was insufficient to allow the samples to exert the desired effects, leading to ID_50_ values > 200 µg/mL.

In light of the above, only data from UAM-EtOH and PLE extractions are presented (Figure S19), expressed in terms of both ID_50_ (represented by bars) and TEAC values (shown as circular markers). PLE extracts consistently showed a better radical scavenging activity for both DPPH and ABTS^+^ radicals, as supported by lower ID_50_ values and higher TEAC values. This evidence could be explained by considering that the content (%) of flavonoids, secoiridoids, and HCA derivatives was higher than that of the UAM-EtOH ones, as these latter ones also contained 20–40% (depending on the cultivar) of triterpenes. These findings suggest that PLE extracts, rich in polar bioactives like phenolic compounds, could be particularly valuable in applications where a potent antioxidant activity is desired, such as in functional foods or nutraceuticals aimed at preventing oxidative-stress-related diseases. Indeed, literature data have demonstrated that, among others, the neuroprotective properties of a plant extract could be attributed to its antioxidant capabilities and ability to mitigate neuroinflammatory processes triggered by free radicals [54]. Thus, in addition to the antioxidant potential, the extracts were assessed for their anti-acetylcholinesterase (AChE) activity, a critical factor in the management of neurodegenerative diseases such as Alzheimer’s disease [55] where cholinergic dysfunction is a hallmark feature. The primary role of AChE is to terminate neuronal transmission and signaling between synapses to prevent ACh dispersal and activation of nearby receptors [56,57]. Using Ellman’s method, modified with a fluorescent probe, the inhibitory activity of the extracts against AChE was measured, with galantamine serving as the positive control [22]. PLE extracts were screened for their AChE inhibitory capacity and the results were compared to those recorded for SFE samples. The rationale was to test and compare mixtures of (poly)phenols and triterpene compounds separately, as UAM extracts showed a similar content to both classes. Indeed, the triterpenes that characterized SFE extracts could be considered effective inhibitors of both enzymes, based on recent literature data [58]. The comparative analysis was performed based on ID_50_ values (µg/mL), which indicate the concentration required to inhibit 50% of AChE activity (Figure S20). Generally, extracts from SFE of all the cultivars proved to be 4- to 12-fold more responsive than the corresponding PLE. Among the SFE samples, a slight difference was observed, with ‘Itrana’ and ‘Leccino’ being the cultivars with the highest AChE activity. Considering that the amount of triterpene acids was comparable among the samples, the higher activity could be ascribed to the higher amount of the triterpene alcohol erythrodiol, whose involvement in neuroprotection has been previously described [59]. Overall, the results demonstrated a clear relationship between the chemical composition of the extracts and their bioactivity. Extracts rich in phenolic compounds and secoiridoids (especially from PLE) exhibited the strongest antioxidant activity, suggesting that these compounds are key contributors to the antioxidant functional properties of olive leaf extracts. Meanwhile, extracts with a higher concentration of fatty acids and triterpenes (especially from SFE) showed moderate antioxidant activities but a very high AChE inhibition, highlighting the diverse range of bioactive compounds that contribute to the overall health benefits of olive leaf by-products. These findings underscore the importance of sequential extractions, choosing the appropriate method, to achieve fractions enriched in specific bioactive compounds, depending on the desired health applications (e.g., antioxidants for functional foods or AChE inhibitors for neuroprotective purposes). Further studies are warranted to investigate the mechanisms behind these activities and to explore their efficacy in in vivo models.

## 4. Conclusions

This study demonstrates the potential of olive leaf by-products from five cultivars (‘Caiazzana’, ‘Carolea’, ‘Itrana’, ‘Leccino’, ‘Frantoio’) as a valuable resource for bioactive compound extraction through a biorefinery perspective. The results show that the chemical profile of the obtained extracts, with regard to both polar and non-polar bioactives, was massively affected by the applied extraction method. This evidence should be carefully taken into account in future experimental designs to modulate the sample chemical complexity better, as extracts differing in phytochemical composition show different bioactivity features. However, scaling up these processes for industrial applications should be guided by a balance between cost efficiency and the preservation of bioactive properties to support the long-term sustainability of olive leaf valorization. In fact, UAM could be the most economically valuable option for small-scale applications due to its ease of use and lower initial investment. On the other hand, SFE and PLE, despite their higher costs, offer an enhanced selectivity and efficiency, making them promising candidates for high-value extract production in the food and nutraceutical industries.

## Figures and Tables

**Table 1:** UHPLC-ESI-QqToF/MS data of the compounds detected in *O. europaea* leaf UAM *n*-hexane and EtOH extracts from the five cultivars under study (Cai = ‘Caiazzana’; Car = ‘Carolea’; Itr = ‘Itrana’; Lec = ‘Leccino’; Fra = ‘Frantoio’). Rt = retention time; RDB = ring and double bond value. The occurrence in each cultivar is highlighted in colored boxes.

	Class	Rt (min)	Tentative Assignment	[M-H]^−^ (*m*/*z*)	Formula	RDB	Error (ppm)		Cai	Car	Itr	Lec	Fra
*n*-hexane extracts	FA	7.983	9-Oxooctadeca-10,12-dienoic acid	293.2127	C_18_H_30_O_3_	4	1.6						
	FA	8.712	Hydroxyoctadecadienoic acid	295.2281	C_18_H_32_O_3_	3	0.8						
	FA	11.462	α-Linolenic acid	277.2177	C_18_H_30_O_2_	4	1.4						
	FA	12.113	Palmitoleic acid	253.2170	C_16_H_30_O_2_	2	−1.2						
	FA	12.481	Linoleic acid	279.2339	C_18_H_32_O_2_	3	3.4						
	FA	13.312	Palmitic acid	255.2338	C_16_H_32_O_2_	1	3.3						
	FA	13.635	Oleic acid	281.2494	C_18_H_34_O_2_	2	2.8						
	T	14.080	Acetyl oleanolic acid	497.3639	C_32_H_50_O_4_	8	0.5						
	FA	14.998	Stearic acid	283.2647	C_18_H_36_O_2_	1	1.6						
EtOH extracts	S	0.288	Hexose hexitol	343.1249	C_12_H_24_O_11_	1	0.9						
	O	0.299	Quinic acid	191.0560	C_7_H_12_O_6_	2	−0.6	*					
	S	0.333	Dihexose	341.1089	C_12_H_22_O_11_	2	1.4						
	O	0.756	Hydroxytyrosol	153.0563	C_8_H_10_O_3_	4	3.8	*					
	O	0.799	Hydroxytyrosol hexoside	315.1086	C_14_H_20_O_8_	5	0.4	*					
	SIr	0.902	Oleoside	389.1099	C_16_H_22_O_11_	6	2.5						
	HCAd	1.332	Caffeic acid hexoside	341.0882	C_15_H_18_O_9_	7	1.2						
	SIr	1.851	Secologanoside	389.1089	C_16_H_22_O_11_	6	3.0						
	GAr	2.322	Benzyl primeveroside	401.1454	C_18_H_26_O_10_	6	0.2						
	SIr	2.536	Elenolic acid hexoside I	403.1252	C_17_H_24_O_11_	6	1.5						
	HCAd	3.000	Ferulic acid hexitolylhexoside	519.1725	C_22_H_32_O_14_	7	1.1						
	SIr	3.285	Elenolic acid hexoside II	403.1251	C_17_H_24_O_11_	6	1.3						
	HCAd	3.292	Ferulic acid hexoside	355.1031	C_16_H_20_O_9_	7	−1.0						
	O	3.367	12-Hydroxyjasmonate sulfate	305.0701	C_12_H_18_O_7_S	4	0.2	*					
	GAr	3.379	Phenetyl primeveroside	415.1615	C_19_H_28_O_10_	6	1.3	*					
	SIr	3.456	Elenolic acid hexoside III	403.1251	C_17_H_24_O_11_	6	1.3						
	Fla	3.745	Apigenin di-*C*-hexoside	593.1520	C_27_H_30_O_15_	13	1.4						
	HCAd	4.369	Campneoside II	639.1938	C_29_H_36_O_16_	12	1.2						
	Fla	4.564	Luteolin dihexoside I	609.1471	C_27_H_30_O_16_	13	1.6	*					
	SIr	4.805	Hydroxyoleuropein	555.1730	C_25_H_32_O_14_	10	1.9						
	Fla	5.177	Luteolin dihexoside II	609.1469	C_27_H_30_O_16_	13	1.3						
	Fla	5.707	Quercetin deoxyhexosylhexoside	609.1473	C_27_H_30_O_16_	13	2.0	*					
	Fla	5.792	Quercetin hexoside	463.0888	C_21_H_20_O_12_	12	1.3						
	Fla	5.983	Luteolin hexoside I	447.0950	C_21_H_20_O_11_	12	3.8	*					
	HCAd	5.985	Verbascoside	623.1987	C_29_H_36_O_15_	12	0.9	*					
	Fla	6.144	Luteolin deoxyhexosylhexoside I	593.1526	C_27_H_30_O_15_	13	2.4						
	Fla	6.571	Eriodictyol hexoside	449.1093	C_21_H_22_O_11_	11	0.8						
	Fla	6.702	Luteolin deoxyhexosylhexoside II	593.1526	C_27_H_30_O_15_	13	2.4						
	SIr	6.794	Oleacein	319.1191	C_17_H_20_O_6_	3	1.2	*					
	HCAd	7.215	Verbascoside der.	667.2260	C_31_H_40_O_16_	12	2.5						
	Fla	7.571	Apigenin hexoside	431.1000	C_21_H_20_O_10_	12	3.8						
	SIr	7.584	Oleuropein hexoside I	701.2314	C_31_H_42_O_18_	11	2.2						
	Fla	7.692	Apigenin deoxyhexosylhexoside I	577.1584	C_27_H_30_O_14_	13	3.7	*					
	Fla	7.824	Luteolin hexoside II	447.0942	C_21_H_20_O_11_	12	2.0	*					
	SIr	7.908	Oleuropein hexoside II	701.2307	C_31_H_42_O_18_	11	1.2	*					
	Fla	7.971	Luteolin dihexoside III	609.1480	C_27_H_30_O_16_	13	3.1						
	Fla	8.033	Luteolin deoxyhexosylhexoside III	593.1519	C_27_H_30_O_16_	13	2.4						
	Fla	8.227	Apigenin deoxyhexosylhexoside II	577.1575	C_27_H_30_O_14_	13	2.1	*					
	Fla	8.394	Diosmetin deoxyhexosylhexoside I	607.1693	C_28_H_32_O_15_	13	4.0						
	Fla	8.396	Diosmetin hexoside	461.1104	C_22_H_22_O_11_	12	3.2						
	SIr	8.404	Oleuropein	539.1789	C_25_H_32_O_13_	10	3.5	*					
	Fla	8.584	Luteolin hexoside III	447.0944	C_21_H_20_O_11_	12	2.5	*					
	Fla	8.782	Diosmetin deoxyhexosylhexoside II	607.1693	C_28_H_32_O_15_	13	4.0	*					
	HCAd	8.860	Osmanthuside B	591.2092	C_29_H_36_O_13_	12	1.5						
	SIr	9.077	*p*-Coumaroyl secologanic acid	535.1477	C_25_H_28_O_13_	12	3.7						
	SIr	9.209	Oleuropein isomer	539.1782	C_25_H_32_O_13_	10	2.2						
	Fla	9.426	Quercetin	301.0356	C_15_H_10_O_7_	11	0.7						
	Fla	9.692	Luteolin	285.0412	C_15_H_10_O_6_	11	2.6	*					
	Fla	9.987	Luteolin deoxyhexosylhexoside der.	979.2748	C_44_H_52_O_25_	19	2.4						
	Fla	10.528	Apigenin deoxyhexosylhexoside der.	963.2796	C_44_H_52_O_24_	19	2.1						
	Fla	10.646	Luteolin *p*-coumaroylhexoside	593.1321	C_30_H_26_O_13_	18	3.4						
	Fla	10.637	Diosmetin deoxyhexosylhexoside der.	993.2905	C_45_H_54_O_25_	19	2.4						
	Fla	10.741	Apigenin	269.0459	C_15_H_10_O_5_	11	1.3	*					
	Fla	11.097	Diosmetin	299.0561	C_16_H_12_O_6_	11	0.1	*					
	T	14.532	Dihydroxy oleanolic acid isomer I	487.3431	C_30_H_48_O_5_	7	0.4						
	T	14.705	Dihydroxy oleanolic acid isomer II	487.3431	C_30_H_48_O_5_	7	0.4						
	T	15.152	Dihydroxy oleanolic acid isomer III	487.3434	C_30_H_48_O_5_	7	1.0						
	T	16.573	Dihydroxy oleanolic acid isomer IV	487.3434	C_30_H_48_O_5_	7	1.0						
	T	16.966	Hydroxy-oxo-oleanenoic acid	469.3320	C_30_H_46_O_4_	8	−0.7						
	T	17.252	Maslinic acid	471.3480	C_30_H_48_O_4_	7	0.0						
	T	19.339	Oleanolic acid	455.3535	C_30_H_48_O_3_	7	0.9	*					
	T	19.614	Ursolic acid	455.3531	C_30_H_48_O_3_	7	0.1						
	T	19.923	Betulinic acid	455.3532	C_30_H_48_O_3_	7	0.3						

FA = fatty acids; Fla = flavonoids; GAr = glycosidic aroma precursors, HCAd = hydroxycinnamic acid derivatives; S = sugars; SIr = secoiridoids; T = triterpene; O = others. * Compounds previously fully characterized [37].

**Table 2:** UHPLC-ESI-QqToF/MS data of the compounds detected in *O. europaea* leaf SFE extracts from the five cultivars under study (Cai = ‘Caiazzana’; Car = ‘Carolea’; Itr = ‘Itrana’; Lec = ‘Leccino’; Fra = ‘Frantoio’). The occurrence is highlighted in colored boxes. Rt = retention time; RDB = ring and double bond value.

Class	Rt (min)	Tentative Assignment	[M-H]^−^ (*m*/*z*)	Formula	RDB	Error (ppm)	Cai	Car	Itr	Lec	Fra
FA	1.136	9-Oxooctadeca-10,12-dienoic acid	293.2127	C_18_H_30_O_3_	4	1.6					
FA	1.482	Hydroxyoctadecadienoic acid	295.2281	C_18_H_32_O_3_	3	0.8					
T	1.845	Maslinic acid	471.3480	C_30_H_48_O_4_	7	−0.2					
T	1.967	Corosolic acid	471.3474	C_30_H_48_O_4_	7	−1.2					
T	3.131	Oleanolic acid	455.3533	C_30_H_48_O_3_	7	0.5					
FA	3.133	α-Linolenic acid	277.2177	C_18_H_30_O_2_	4	1.4					
T	3.472	Ursolic acid	455.3530	C_30_H_48_O_3_	7	−0.2					
FA	3.615	Palmitoleic acid	253.2170	C_16_H_30_O_2_	2	−1.2					
T	3.637	Betulinic acid	455.3525	C_30_H_48_O_3_	7	−1.3					
FA	3.988	Linoleic acid	279.2339	C_18_H_32_O_2_	3	3.4					
FA	4.657	Palmitic acid	255.2338	C_16_H_32_O_2_	1	3.3					
FA	4.928	Oleic acid	281.2494	C_18_H_34_O_2_	2	2.8					
T	5.212	Acetyl oleanolic acid	497.3639	C_32_H_50_O_4_	8	0.5					
FA	5.994	Stearic acid	283.2647	C_18_H_36_O_2_	1	1.6					

FA = fatty acids; T = triterpene.

**Table 3:** GC-MS data of the compounds detected in *O. europaea* leaf SFE extracts from the five cultivars under study (Cai = ‘Caiazzana’; Car = ‘Carolea’; Itr = ‘Itrana’; Lec = ‘Leccino’; Fra = ‘Frantoio’). The occurrence is highlighted in colored boxes. Rt = retention time.

Class	Rt (min)	Tentative Assignment	MW (*m*/*z*)	Formula	Similarity (%)	Cai	Car	Itr	Lec	Fra
Sq	15.756	Globulol	222	C_15_H_26_O	84					
FA	19.337	Palmitic acid	256	C_16_H_32_O_2_	94					
FA	21.811	Linoleic acid	280	C_18_H_32_O_2_	91					
FAl	21.916	Octadecadienal	264	C_18_H_32_O	91					
FA	21.936	Linolenic acid	278	C_18_H_30_O_2_	91					
T	31.872	Squalene	410	C_30_H_50_	92					
H	32.766	Heneicosane	296	C_21_H_44_	92					
H	35.357	Tetratriacontane	478	C_34_H_70_	91					
V	35.961	α-Tocopherol	430	C_29_H_50_O_2_	73					
V	36.033	Ttocopherol isomer	430	C_29_H_50_O_2_	-					
H	38.017	Docosane	310	C_22_H_46_	87					
St	38.675	β-Sitosterol	414	C_29_H_50_O	86					
T	39.273	Lupeol I	426	C_30_H_50_O	78					
T	39.967	Lupeol II	426	C_30_H_50_O	82					
T	44.448	Erythrodiol	442	C_30_H_50_O_2_	76					
T	45.335	Urs-12-en-28-ol	426	C_30_H_50_O	76					

FA = fatty acid; FAl = fatty aldehydes; H = hydrocarbons; Sq = sesquiterpenes; St = sterols; T = triterpene; V = vitamins.

**Table 4:** UHPLC-ESI-QqToF/MS data of the compounds detected in *O. europaea* leaf PLE extracts from the five cultivars under study (Cai = ‘Caiazzana’; Car = ‘Carolea’; Itr = ‘Itrana’; Lec = ‘Leccino’; Fra = ‘Frantoio’). The occurrence is highlighted in colored boxes. Rt = retention time; RDB = ring and double bond value.

Class	Rt (min)	Tentative Assignment	[M-H]^−^ (*m*/*z*)	Formula	RDB	Error (ppm)	Cai	Car	Itr	Lec	Fra
S	0.293	Hexose hexitol	343.1249	C_12_H_24_O_11_	1	0.9					
S	0.322	Dihexose	341.1089	C_12_H_22_O_11_	2	1.4					
SIr	0.499	Oleoside	389.1099	C_16_H_22_O_11_	6	2.5					
O	0.514	Hydroxytyrosol hexoside	315.1086	C_14_H_20_O_8_	5	2.4					
HCAd	0.710	Caffeic acid hexoside	341.0882	C_15_H_18_O_9_	7	1.2					
SIr	0.947	Secologanoside	389.1089	C_16_H_22_O_11_	6	3.0					
O	1.205	12-Hydroxyjasmonate sulfate	305.0701	C_12_H_18_O_7_S	4	2.5					
GAr	1.330	Benzyl primeveroside	401.1454	C_18_H_26_O_10_	6	0.2					
SIr	1.462	Elenolic acid hexoside I	403.1252	C_17_H_24_O_11_	6	1.5					
HCAd	1.652	Ferulic acid hexitolylhexoside	519.1725	C_22_H_32_O_14_	7	1.1					
SIr	2.246	Elenolic acid hexoside II	403.1251	C_17_H_24_O_11_	6	1.3					
Fla	2.279	Apigenin di-*C*-hexoside	593.1520	C_27_H_30_O_15_	13	1.4					
GAr	2.354	Phenetyl primeveroside	415.1615	C_19_H_28_O_10_	6	1.3					
HCAd	3.049	Campneoside II	639.1938	C_29_H_36_O_16_	12	1.2					
Fla	3.155	Luteolin dihexoside I	609.1471	C_27_H_30_O_16_	13	1.6					
SIr	3.716	Hydroxyoleuropein	555.1730	C_25_H_32_O_14_	10	1.9					
Fla	3.931	Luteolin dihexoside II	609.1469	C_27_H_30_O_16_	13	1.3					
SIr	4.348	Oleuropein hexoside I	701.2304	C_31_H_42_O_18_	11	0.8					
Fla	4.453	Quercetin deoxyhexosylhexoside	609.1473	C_27_H_30_O_16_	13	2.0					
Fla	4.575	Quercetin hexoside	463.0888	C_21_H_20_O_12_	12	1.3					
Fla	4.731	Luteolin hexoside I	447.0950	C_21_H_20_O_11_	12	3.8					
Fla	4.794	Luteolin deoxyhexosylhexoside I	593.1519	C_27_H_30_O_16_	13	4.9					
HCAd	4.800	Verbascoside	623.1987	C_29_H_36_O_15_	12	0.9					
Fla	5.231	Luteolin deoxyhexosylhexoside II	593.1526	C_27_H_30_O_15_	13	2.4					
SIr	5.691	Verbascoside derivative	667.2260	C_31_H_40_O_16_	12	2.5					
SIr	5.902	Oleuropein hexoside II	701.2314	C_31_H_42_O_18_	11	2.2					
Fla	5.903	Apigenin deoxyhexosylhexoside I	577.1584	C_27_H_30_O_14_	13	3.7					
Fla	5.957	Apigenin hexoside	431.1000	C_21_H_20_O_10_	12	3.8					
Fla	6.054	Luteolin hexoside II	447.0942	C_21_H_20_O_11_	12	2.0					
Fla	6.072	Luteolin deoxyhexosylhexoside III	593.1519	C_27_H_30_O_16_	13	2.4					
Fla	6.099	Luteolin dihexoside III	609.1480	C_27_H_30_O_16_	13	3.1					
SIr	6.301	Oleuropein hexoside III	701.2307	C_31_H_42_O_18_	11	1.2					
Fla	6.301	Apigenin deoxyhexosylhexoside II	577.1575	C_27_H_30_O_14_	13	2.1					
Fla	6.524	Diosmetin hexoside	461.1104	C_22_H_22_O_11_	12	3.2					
SIr	6.704	Oleuropein	539.1789	C_25_H_32_O_13_	10	3.5					
Fla	6.748	Luteolin hexoside III	447.0944	C_21_H_20_O_11_	12	2.5					
Fla	6.841	Diosmetin deoxyhexosylhexoside	607.1693	C_28_H_32_O_15_	13	4.0					
SIr	7.393	*p*-Coumaroyl secologanic acid	535.1477	C_25_H_28_O_13_	12	3.7					
SIr	7.626	Oleuropein isomer	539.1782	C_25_H_32_O_13_	10	2.2					
Fla	7.952	Quercetin	301.0356	C_15_H_10_O_7_	11	0.7					
Fla	8.350	Luteolin	285.0412	C_15_H_10_O_6_	11	2.6					
Fla	8.940	Luteolin deoxyhexosylhexoside der	979.2748	C_44_H_52_O_25_	19	2.4					
Fla	10.049	Apigenin deoxyhexosylhexoside der	963.2796	C_44_H_52_O_24_	19	2.1					
Fla	10.097	Luteolin *p*-coumaroylhexoside	593.1321	C_30_H_26_O_13_	18	3.4					
Fla	10.122	Apigenin	269.0458	C_15_H_10_O_5_	11	0.9					
Fla	10.275	Diosmetin deoxyhexosylhexoside der	993.2905	C_45_H_54_O_25_	19	2.4					
Fla	10.748	Diosmetin	299.0565	C_16_H_12_O_6_	11	1.3					

Fla = flavonoids; GAr = glycosidic aroma precursors, HCAd = hydroxycinnamic acid derivatives; S = sugars; SIr = secoiridoids; O = others.

## Data Availability

The original contributions presented in this study are included in the article/Supplementary Materials. Further inquiries can be directed to the corresponding author.

## Supplementary Materials

The following supporting information can be downloaded at: https://www.mdpi.com/article/10.3390/foods14020297/s1, Figure S1: Total extraction yields (%) by scCO_2_ of samples collected every 20 min up to 200 min for the five olive cultivars under study (Cai = ‘Caiazzana’, Car = ‘Carolea’, Itr = ‘Itrana’, Lec = ‘Leccino’, Fra = ‘Frantoio’); Figure S2: Linear gradient of water (A) and acetonitrile (B), both with 0.1% formic acid, applied to the investigated extracts. At the end of each time program, the starting conditions were restored and the system was re-equilibrated for 2 min. The flow rate was 0.5 mL/min, and the injection volume was 2.0 µL; Figure S3: (A) TOF-MS/MS spectrum of the hypothesized acetyl oleanolic acid, detected in samples from UAM in *n*-hexane extraction, and (B) its fragmentation pathway (theoretical *m*/*z* values are reported below each structure); Figure S4: Free fatty acids composition (%) of leaf extracts obtained by UAM in *n*-hexane from the five olive cultivars under study (Cai = ‘Caiazzana’, Car = ‘Carolea’, Itr = ‘Itrana’, Lec = ‘Leccino’, Fra = ‘Frantoio’); Figure S5: TOF-MS/MS spectra of (A) dihexose; (B) hexose hexitol; (C) benzyl primeveroside; Figure S6: TOF-MS/MS spectra of (A) caffeic acid hexoside; (B) ferulic acid hexitolylhexoside; (C) ferulic acid hexoside; (D) campneoside II; (E) osmanthuside B; (F) verbascoside derivative at 7.215 min; Figure S7: TOF-MS/MS spectra of (A) oleoside; (B) secologanoside; (C) a representative elenolic acid hexoside; (D) hydroxyoleuropein; (E) oleuropein isomer; (F) *p*-coumaroyl secologanic acid; Figure S8: TOF-MS/MS spectra of apigenin glycosides: (A) apigenin di-*C*-hexoside; (B) apigenin hexoside; (C) apigenin deoxyhexosylhexoside derivative; Figure S9: TOF-MS/MS spectra of luteolin glycosides: (A) a representative luteolin dihexoside II; (B) luteolin deoxyhexosylhexoside I; (C) luteolin deoxyhexosylhexoside II; (D) luteolin dihexoside III; (E) luteolin deoxyhexosylhexoside derivative; (F) luteolin p-coumaroylhexoside; Figure S10: TOF-MS/MS spectra of (A) diosmetin deoxyhexosylhexoside I, (B) its derivative, and of (C) diosmetin hexoside; Figure S11: TOF-MS/MS spectra of flavonoids other than flavone: (A) quercetin hexoside, (B) quercetin aglycone, and (C) eriodictyol hexoside; Figure S12: TOF-MS/MS spectra of (A) a representative dihydroxy oleanolic acid isomer, (B) hydroxy-oxo-oleanenoic acid, (C) maslinic acid, and (D) oleanolic acid. In the grey box, extracted ion chromatograms (XICs) for the detected triterpenes are reported; Figure S13: (A) Hierarchical cluster of the five cultivars (Cai = ‘Caiazzana’, Car = ‘Carolea’, Itr = ‘Itrana’, Lec = ‘Leccino’, Fra = ‘Frantoio’). (B) Heatmap analyses of flavonoids in UAM EtOH extracts (Api = apigenin; Dio = diosmetin; Eri = eriodictyol; Lut = luteolin; Que = quercetin; der = derivatives). (C) Scatter plots of luteolin and its most abundant hexoside relative abundance (%) in the five alcoholic extracts (● luteolin, luteolin hexoside I, ● luteolin hexoside II, ▲ luteolin hexoside III); Figure S14: Venn diagram of metabolite identification by GC-MS and LC-MS tools. The chemical structures of the main compounds are also depicted; Figure S15: GC-EI/MS spectra of (A) α-tocopherol and (B) its isomer; Figure S16: Relative amount (%) of compound classes detected in leaf extracts obtained by SFE/GC-MS from the five olive cultivars under study (Cai = ‘Caiazzana’, Car = ‘Carolea’, Itr = ‘Itrana’, Lec = ‘Leccino’, Fra = ‘Frantoio’). FA = fatty acids; FAl = fatty aldehydes; H = hydrocarbons; Sq = sesquiterpenes; St = sterols; T = triterpenes; V = vitamins; Figure S17: GC-EI/MS spectra of lupeol isomer (A) I and (B) II; Figure S18: (A) Representative total ion current chromatogram (TIC) of a PLE extract. Compounds are labeled with different colors, based on the class they belong to (the same colors are used for histograms in panel B). (B) Relative abundance (%) of the six metabolite classes under study in each cultivar (Cai = ‘Caiazzana’, Car = ‘Carolea’, Itr = ‘Itrana’, Lec = ‘Leccino’, Fra = ‘Frantoio’); Figure S19: (A) Radical Scavenging Capacity (ID50 and TEAC values) toward (A) DPPH^•^ and (B) ABTS^•+^ of PLE and UAM extracts from the five olive cultivars under study (Cai = ‘Caiazzana’, Car = ‘Carolea’, Itr = ‘Itrana’, Lec = ‘Leccino’, Fra = ‘Frantoio’). ID50 higher than 200 µg/mL are not reported; Figure S20: Anti-acetylcholinesterase activity of PLE and SFE extracts from the five olive cultivars under study (Cai = ‘Caiazzana’, Car = ‘Carolea’, Itr = ‘Itrana’, Lec = ‘Leccino’, Fra = ‘Frantoio’). Galantamine (Gal) was used as the positive standard; Table S1: Extraction yields (%) of crude extracts obtained from olive leaves of five different cultivars (Cai = ‘Caiazzana’, Car = ‘Carolea’, Itr = ‘Itrana’, Lec = ‘Leccino’, Fra = ‘Frantoio’), by applying ultrasound-assisted maceration (UAM), pressurized liquid extraction (PLE), and supercritical fluid extraction (SFE).
