# Supplementary material for: Recovery of Bioactive Constituents from Olive Leaf Pruning Waste of Five Different Cultivars: A Comparison of Green Extraction Techniques to Maximize Health Benefits
Source: Foods. 2025 Jan 17;14(2):297. doi: 10.3390/foods14020297 (PMC11765081; doi:10.3390/foods14020297)
Supplement: Supplementary file 1 [file foods-14-00297-s001.zip › foods-3414149-supplementary.pdf]

Article

Recovery of Bioactive Constituents from Olive Leaf Pruning Waste of Five Different Cultivars: A Comparison of Green Extraction Techniques to Maximize Health Benefits

Hamid Mushtaq [1], Simona Piccolella [1,*], Jose A. Mendiola [2], Lidia Montero [2], Elena Ibáñez [2] and Severina Pacifico [1]

Supplementary Materials

Table S1. Extraction yields (%) of crude extracts obtained from olive leaves of fivedifferent cultivars (Cai = 'Caiazzana', Car = 'Carolea', Itr = 'Itrana', Lec = 'Leccino', Fra = 'Frantoio'), by applying Ultrasound-Assisted Maceration (UAM), Pressurized Liquid Extraction (PLE), and Supercritical Fluid Extraction (SFE).

	**UAM (*n*-hex)**	**UAM (EtOH)**	**SFE**	**PLE**
**Cai**	2.5 %	30.5 %	1.9 %	32.5 %
**Car**	2.3 %	22.7 %	2.0 %	31.5 %
**Itr**	2.3 %	34.6 %	1.9 %	32.7 %
**Lec**	1.9 %	38.8 %	2.0 %	33.7 %
**Fra**	2.8 %	39.3 %	1.9 %	34.9 %

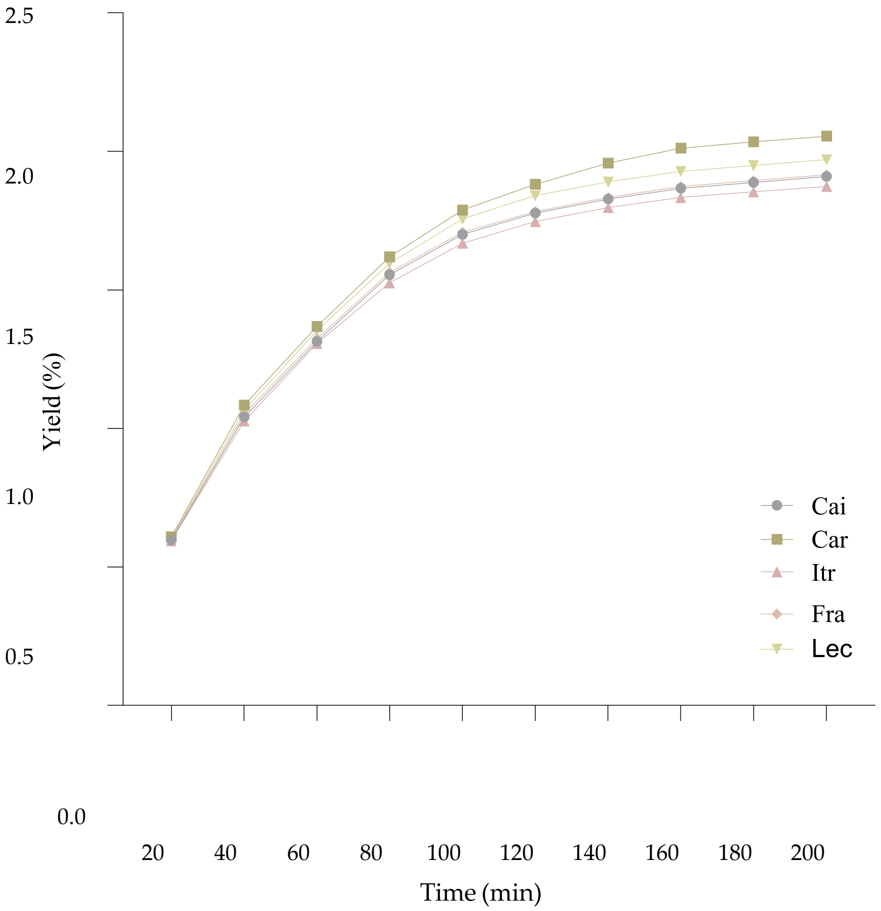

Figure S1. Total extraction yields (%) by $scCO_2$ of samples collected every 20 min up to 200 min for the five olive cultivars under study (Cai = 'Caiazzana', Car = 'Carolea', Itr = 'Itrana', Lec = 'Leccino', Fra = 'Frantoio').

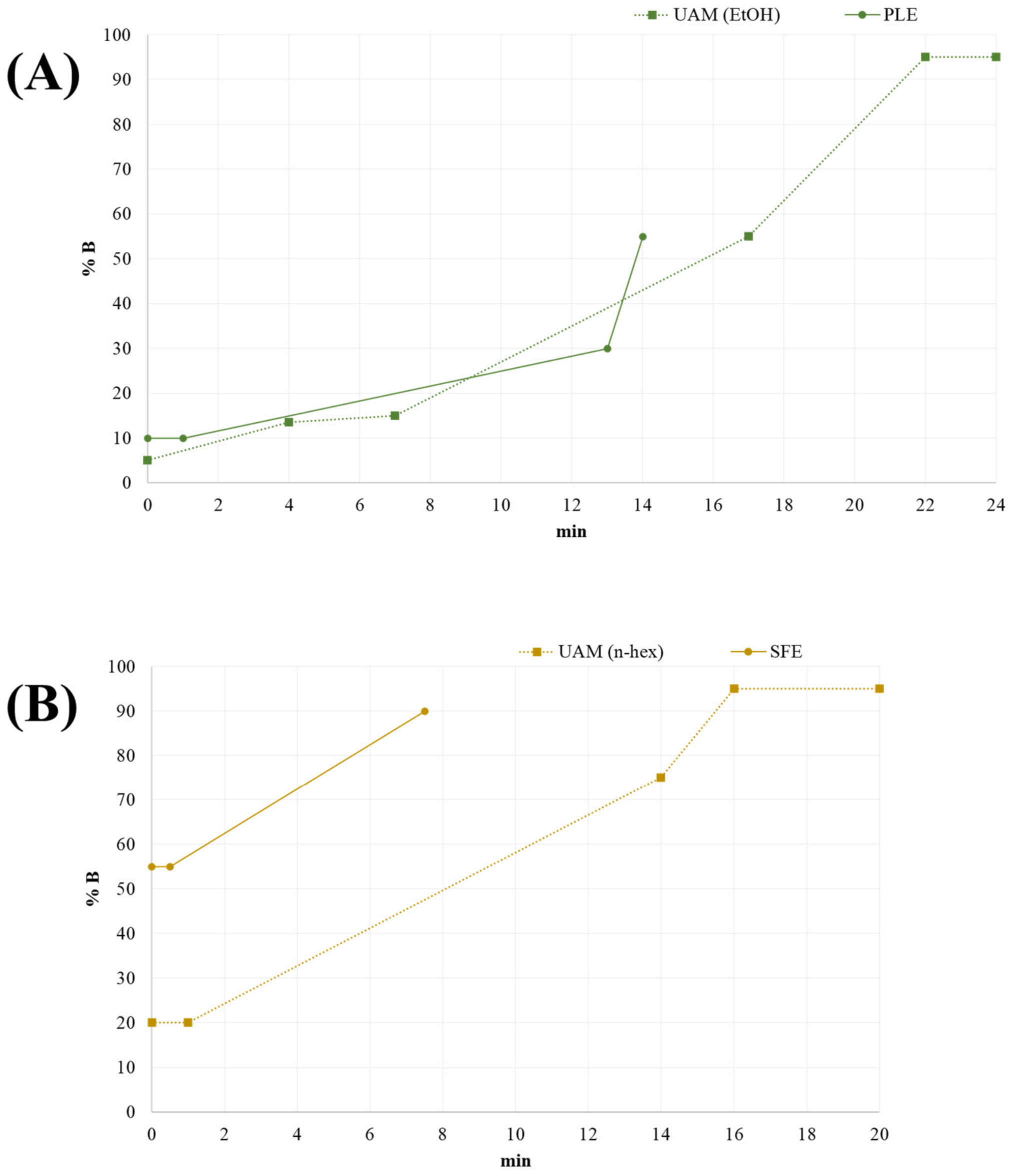

Figure S2. Linear gradient of water (A) and acetonitrile (B), both with 0.1% formicacid, applied to the investigated extracts. At the end of each time program, the starting conditions were restored and the system was re-equilibrated for 2 min. Theflow rate was 0.5 mL/min, and the injection volume was 2.0 μL.

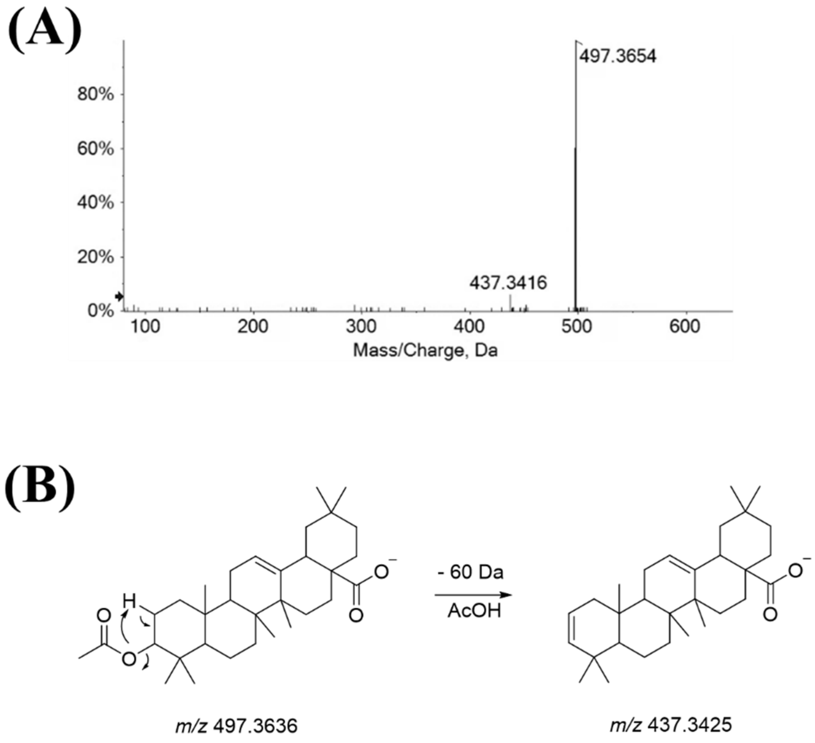

Figure S3. (A) TOF-MS/MS spectrum of the hypothesized acetyl oleanolic acid, detected in samples from UAM in *n*-hexane extraction, and (B) its fragmentation pathway (theoretical *m*/*z* values are reported below each structure).

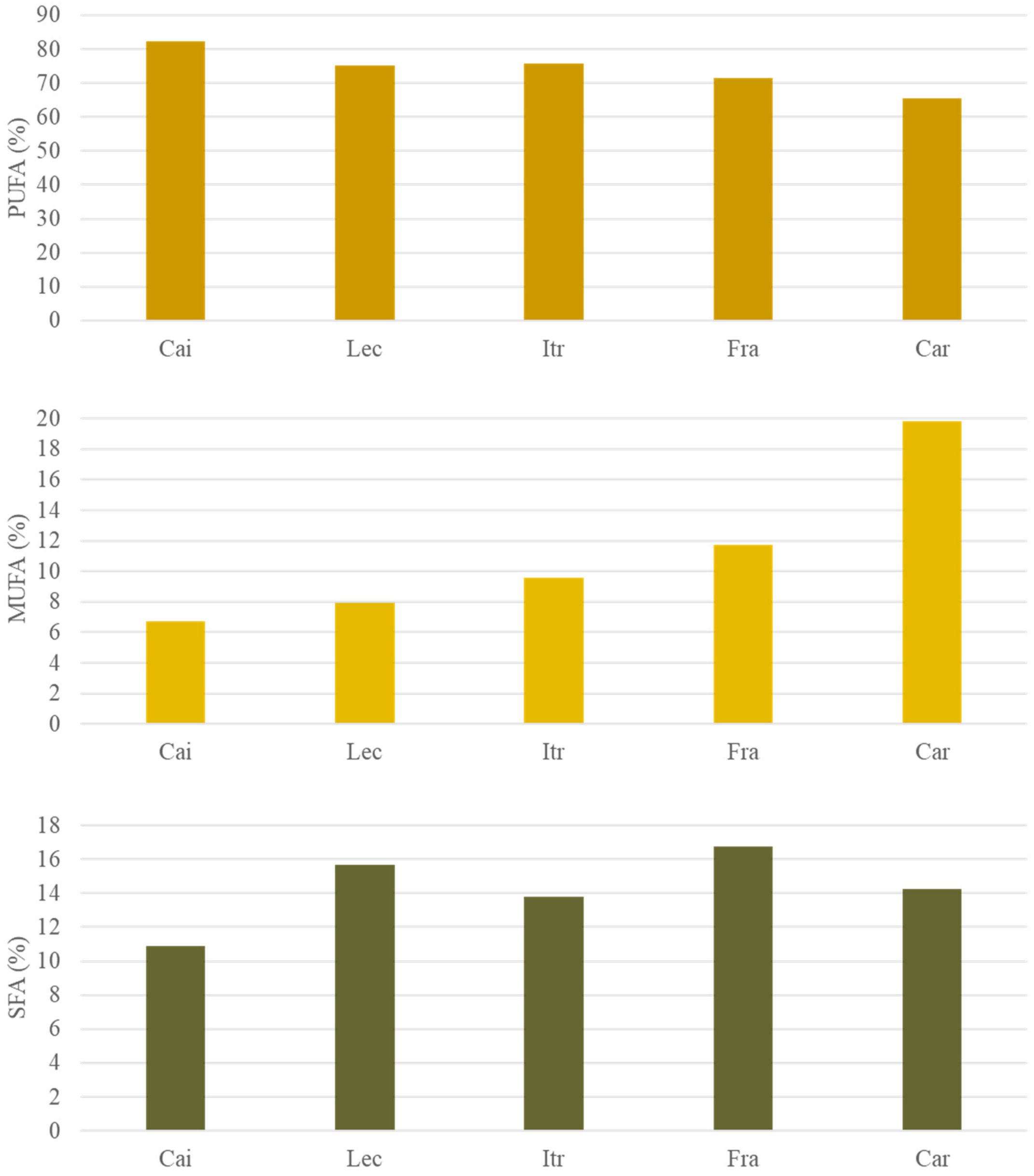

Figure S4. Free fatty acids composition (%) of leaf extracts obtained by UAM in *n*-hexane from the five olive cultivars under study (Cai = 'Caiazzana', Car = 'Carolea', Itr = 'Itrana', Lec = 'Leccino', Fra = 'Frantoio').

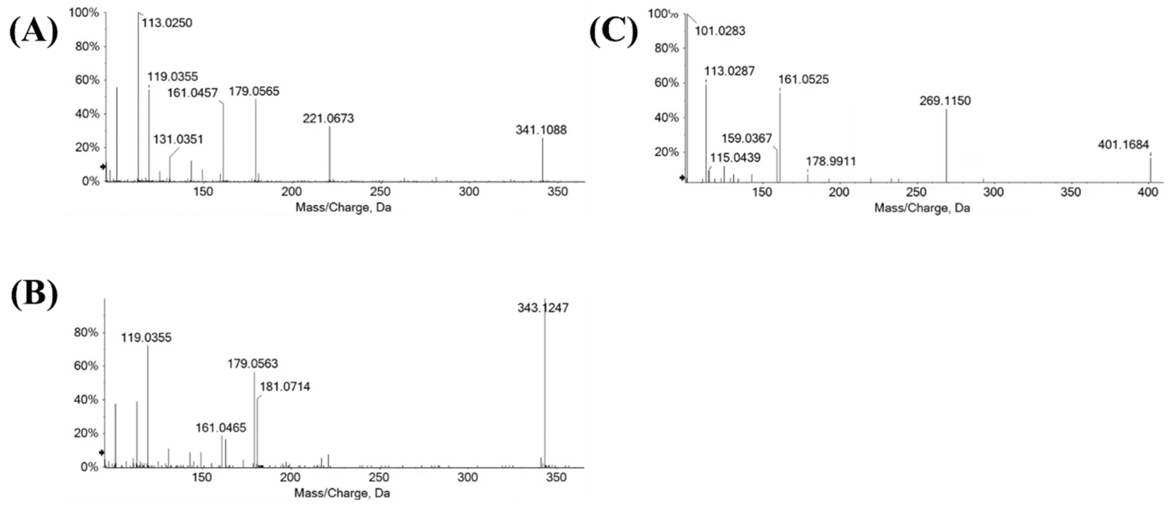

Figure S5. TOF-MS/MS spectra of (A) dihexose; (B) hexose hexitol; (C) benzyl primeveroside.

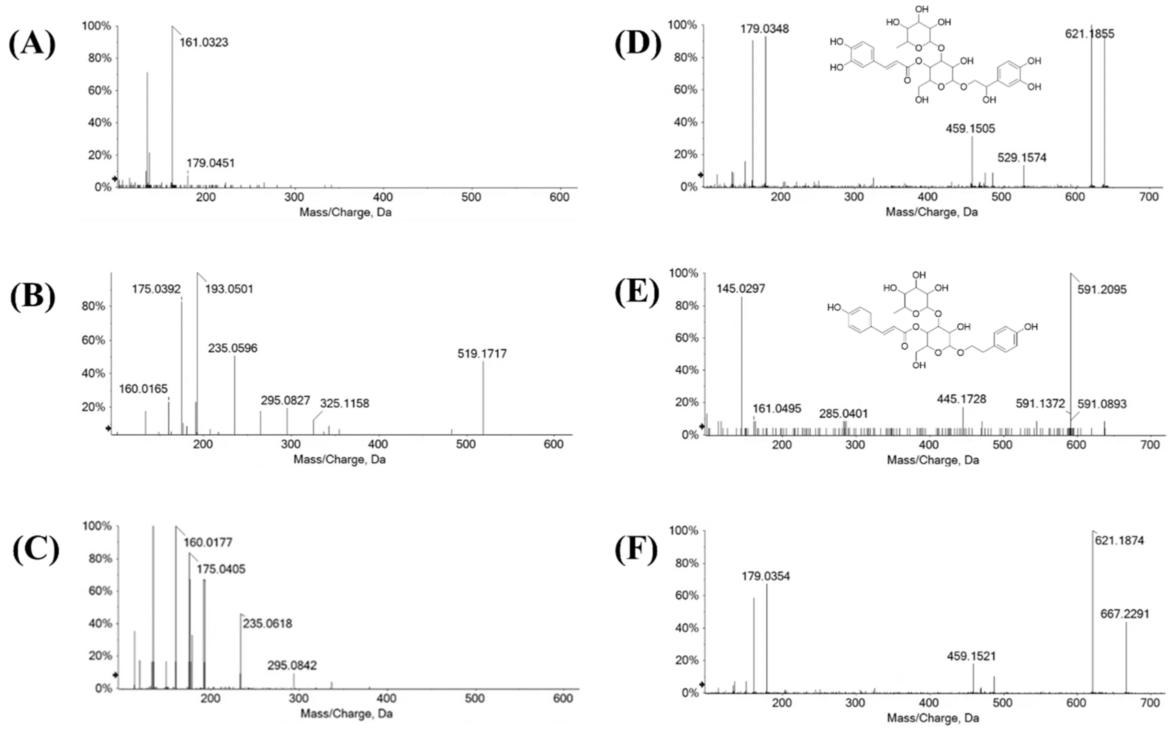

Figure S6. TOF-MS/MS spectra of (A) caffeic acid hexoside; (B) ferulic acid hexitolylhexoside; (C) ferulic acid hexoside; (D) campneoside II; (E) osmanthuside B; (F) verbascoside derivative at 7.215 min.

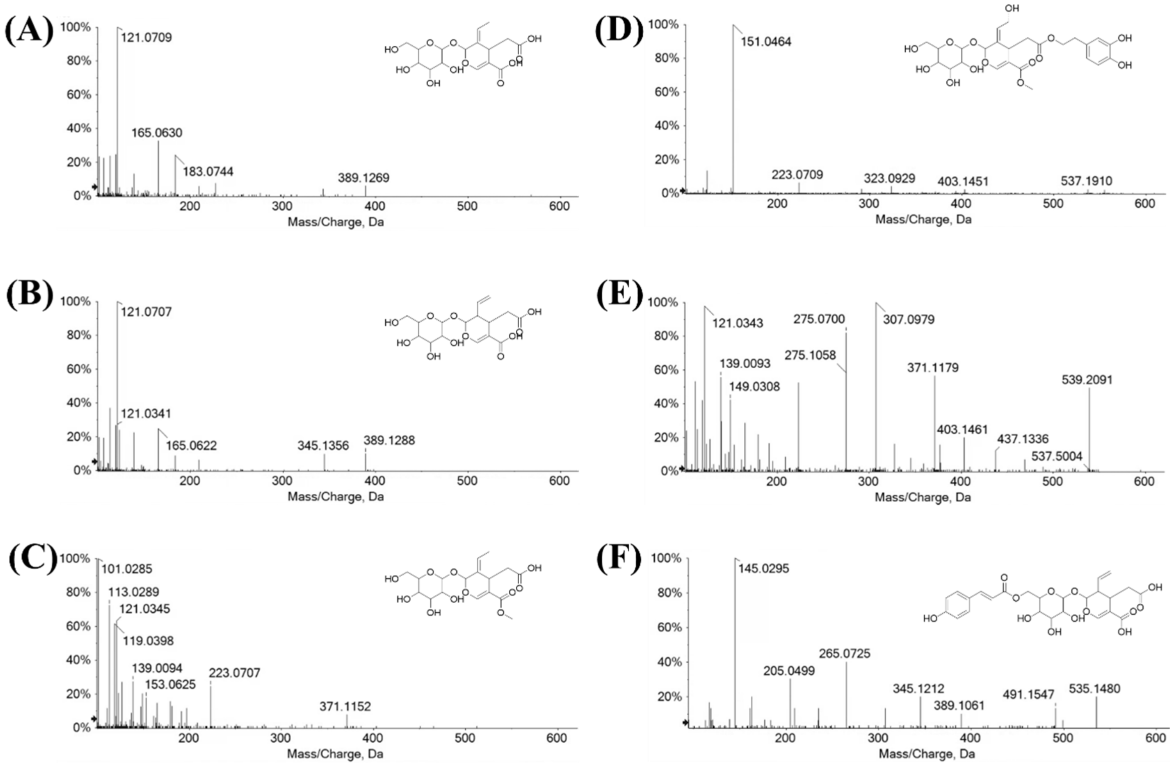

Figure S7. TOF-MS/MS spectra of (A) oleoside; (B) secologanoside; (C) a representative elenolic acid hexoside; (D) hydroxyoleuropein; (E) oleuropein isomer; (F) *p*-coumaroyl secologanic acid.

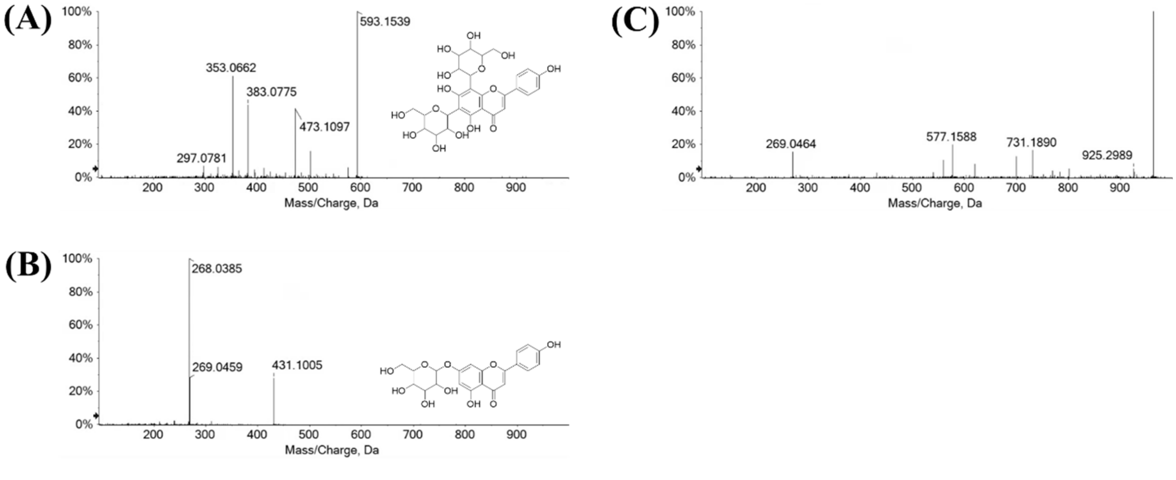

Figure S8. TOF-MS/MS spectra of apigenin glycosides: (A) apigenin di-*C*-hexoside; (B) apigenin hexoside; (C) apigenin deoxyhexosylhexoside derivative.

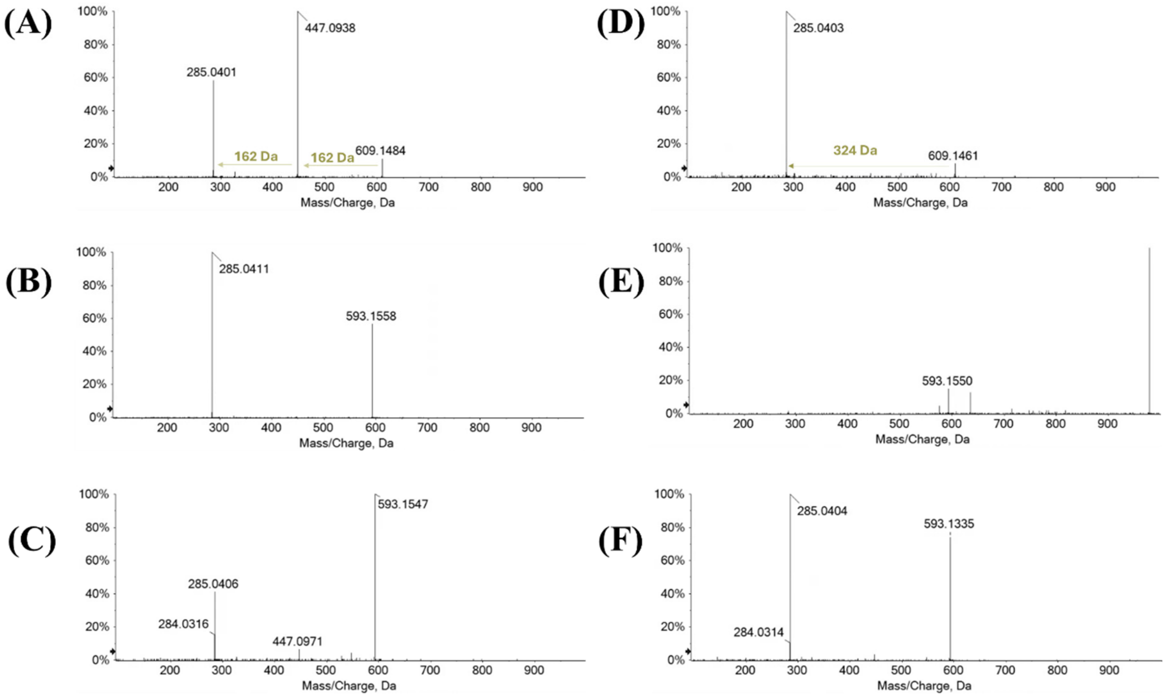

Figure S9. TOF-MS/MS spectra of luteolin glycosides: (A) a representative luteolin dihexoside II; (B) luteolin deoxyhexosylhexoside I; (C) luteolin deoxyhexosylhexoside II; (D) luteolin dihexoside III; (E) luteolin deoxyhexosylhexoside derivative; (F) luteolin p-coumaroylhexoside.

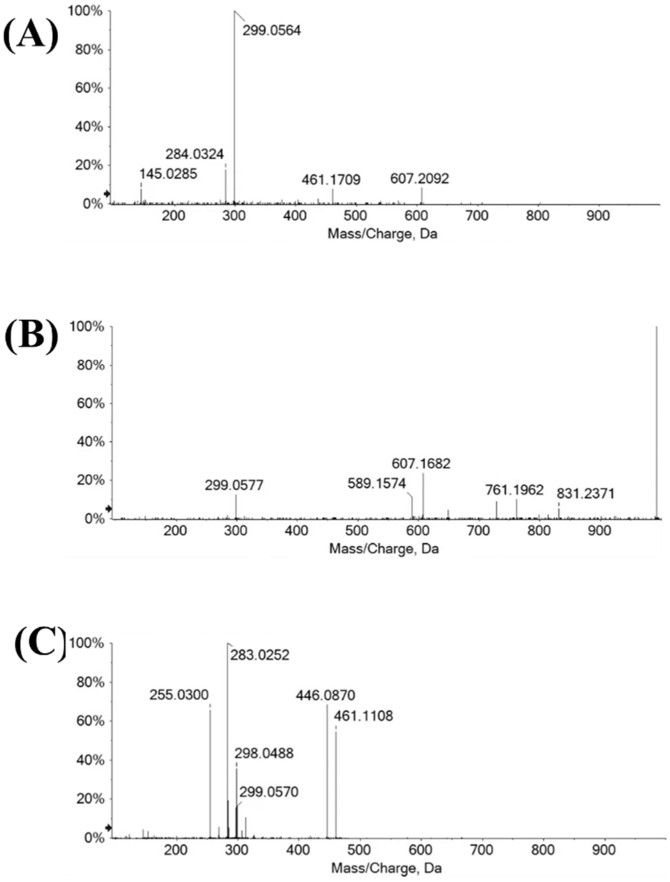

Figure S10. TOF-MS/MS spectra of (A) diosmetin deoxyhexosylhexoside I, (B) itsderivative, and of (C) diosmetin hexoside.

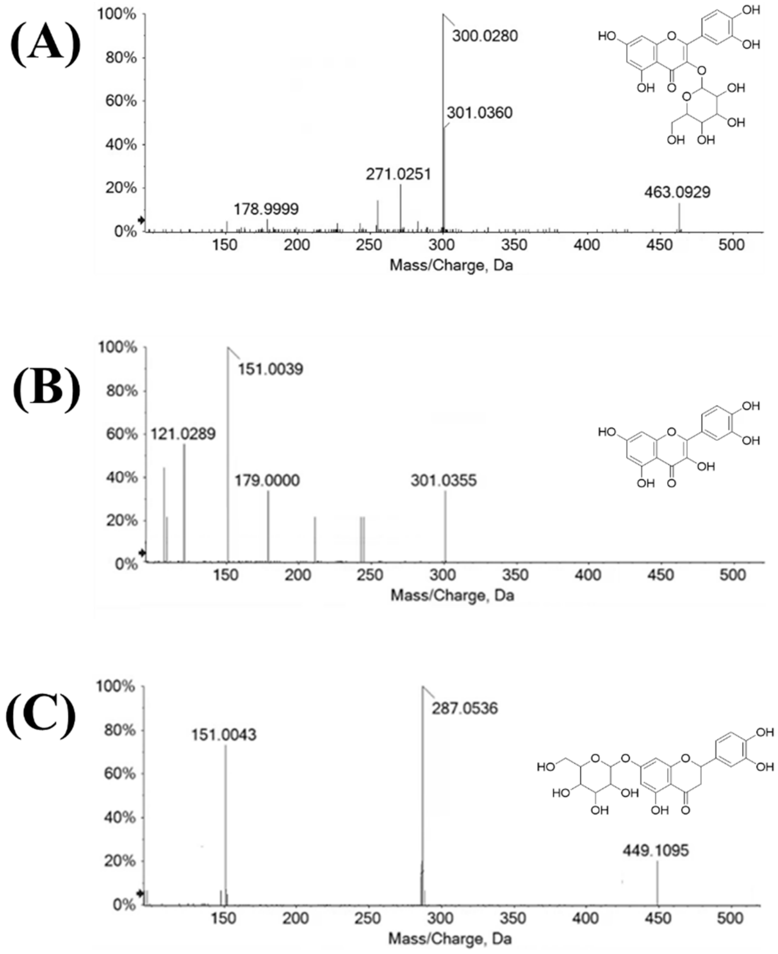

Figure S11. TOF-MS/MS spectra of flavonoids other than flavone: (A) quercetin hexoside, (B) quercetin aglycone, and (C) eriodictyol hexoside.

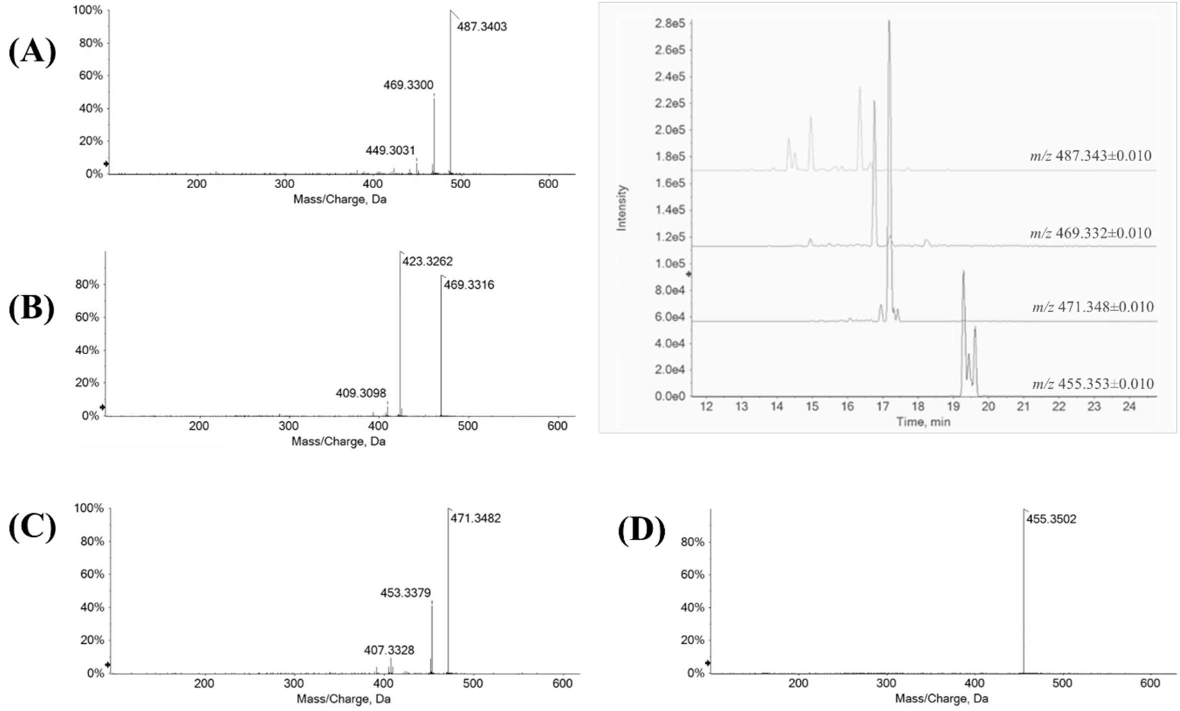

Figure S12. TOF-MS/MS spectra of (A) a representative dihydroxy oleanolic acid isomer, (B) hydroxy-oxo-oleanenoic acid, (C) maslinic acid, and (D) oleanolic acid.In the grey box extracted ion chromatograms (XICs) for

the detected triterpenes arereported.

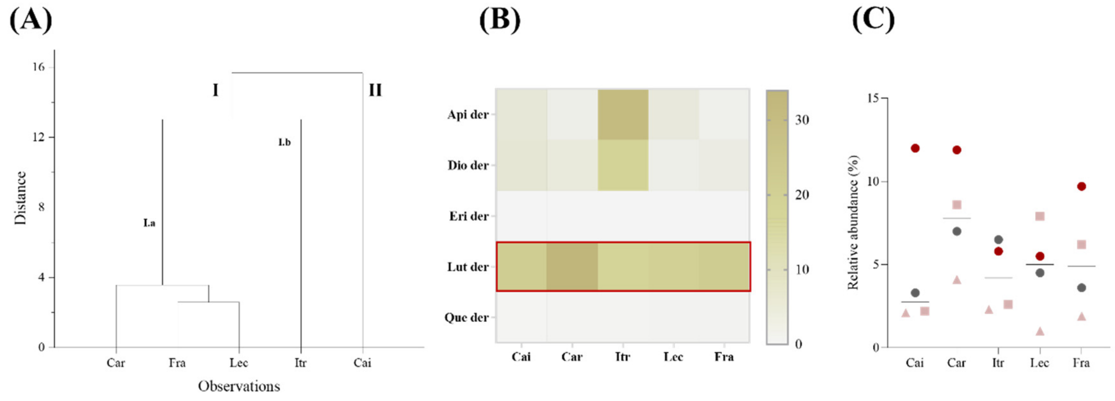

Figure S13. (A) Hierarchical cluster of the five cultivars (Cai = 'Caiazzana', Car = 'Carolea', Itr = 'Itrana', Lec = 'Leccino', Fra = 'Frantoio'). (B) Heatmap analyses of flavonoids in UAM EtOH extracts (Api = apigenin; Dio = diosmetin; Eri = eriodictyol; Lut = luteolin; Que = quercetin; der = derivatives). (C) Scatter plots of luteolin and its most abundant hexoside relative abundance (%) in the five alcoholic extracts (● luteolin, luteolin hexoside I, ● luteolin hexoside II, ▲ luteolin hexoside III).

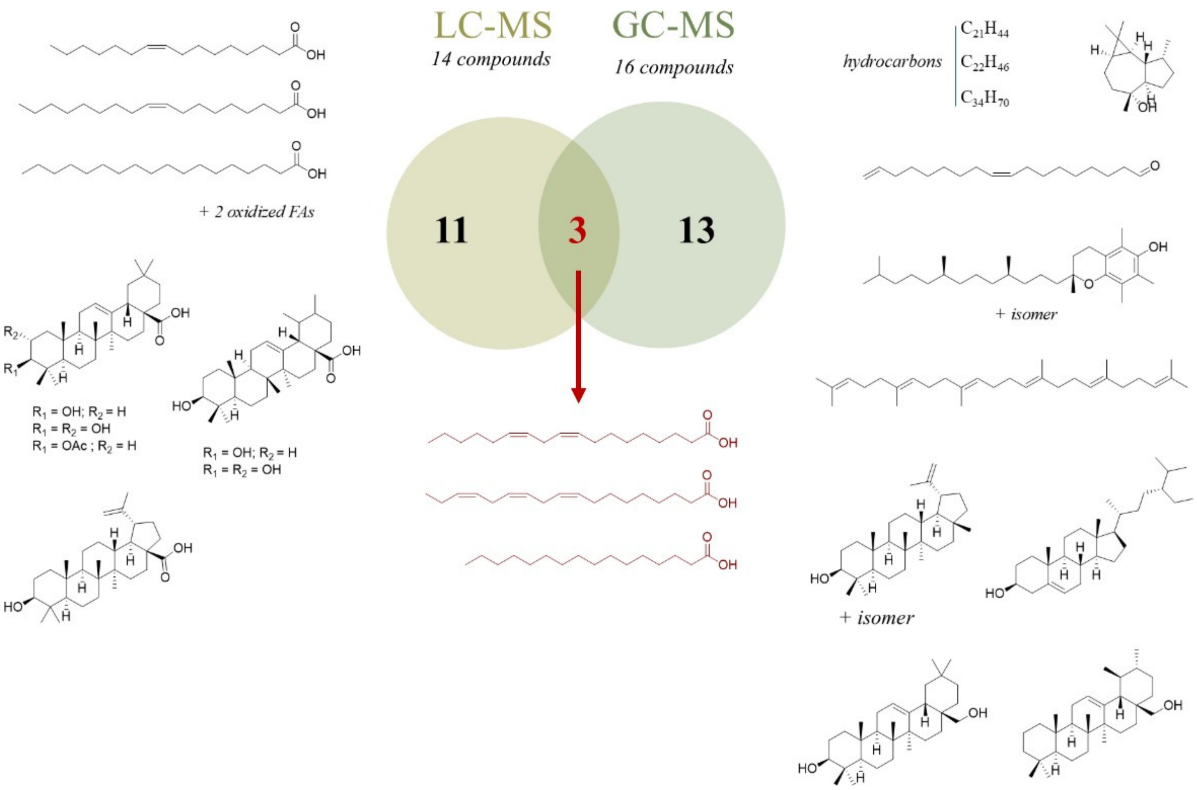

Figure S14. Venn diagram of metabolite identification by GC-MS and LC-MS tools.The chemical structures of the main compounds are also depicted.

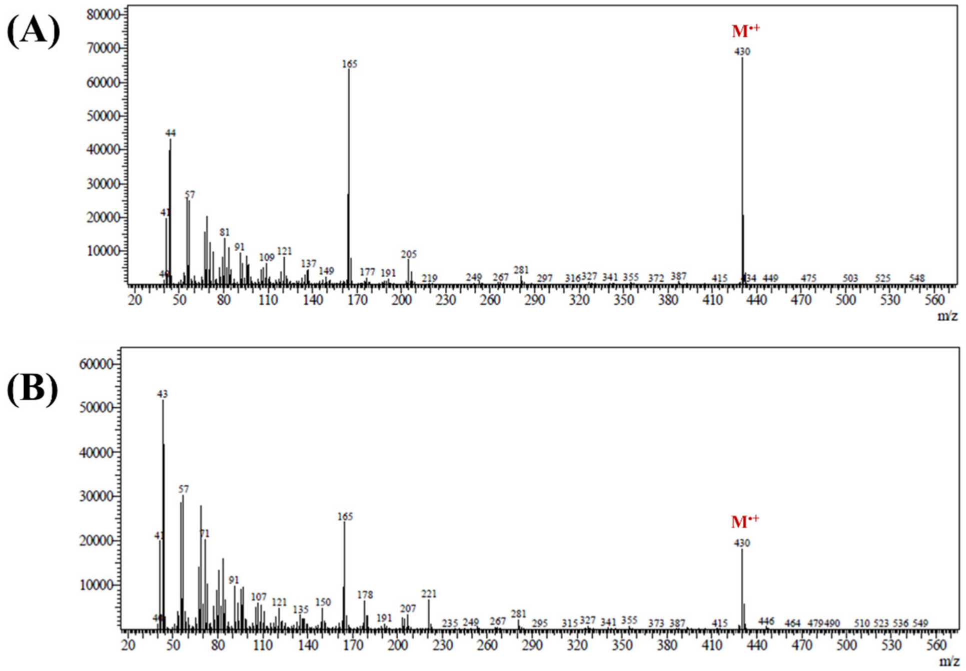

Figure S15. GC-EI/MS spectra of (A) α-tocopherol and (B) its isomer.

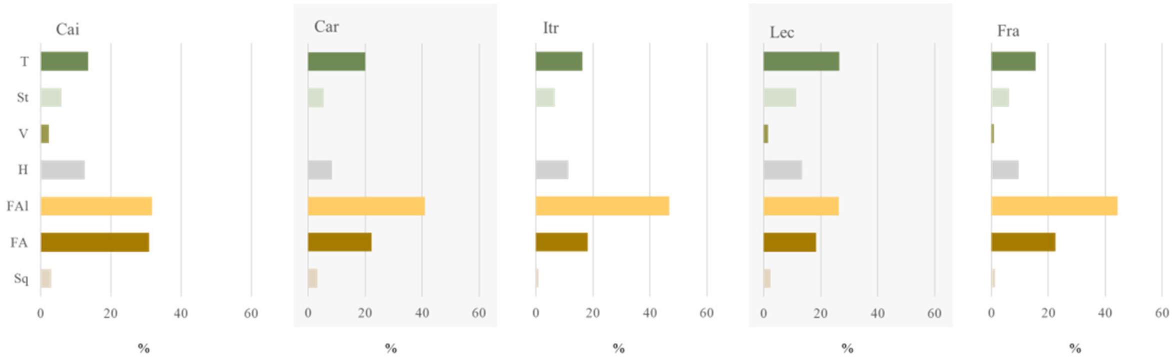

Figure S16. Relative amount (%) of compound classes detected in leaf extracts obtained by SFE/GC-MS from the five olive cultivars under study (Cai = 'Caiazzana', Car = 'Carolea', Itr = 'Itrana', Lec = 'Leccino', Fra = 'Frantoio'). FA = fatty acids; Fal =fatty aldehydes; H = hydrocarbons; Sq = sesquiterpenes; St = sterols; T = triterpenes; V = vitamins.

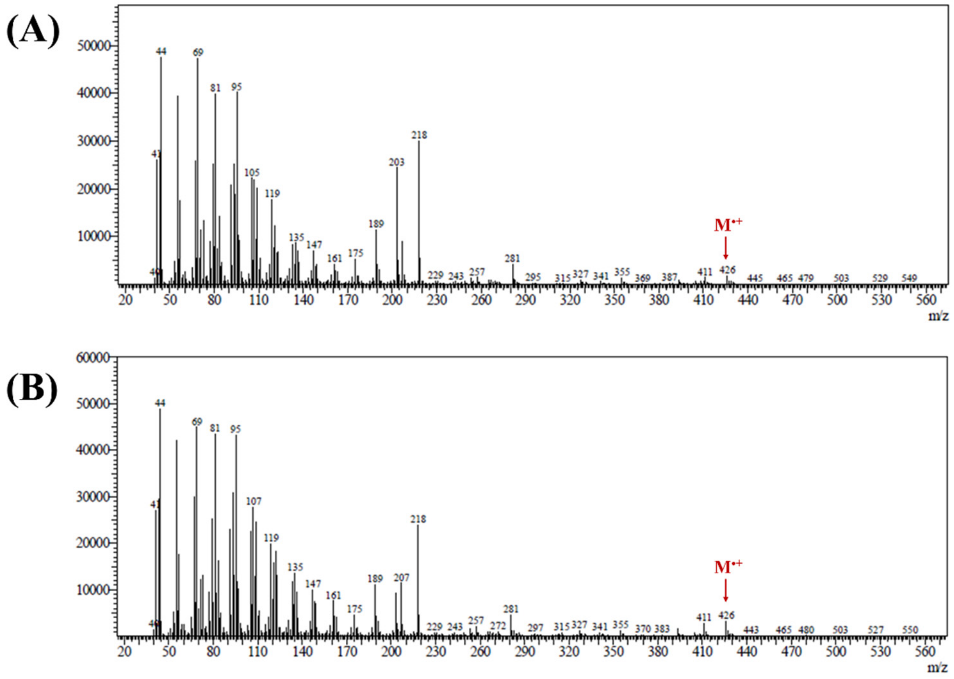

Figure S17. GC-EI/MS spectra of lupeol isomer (A) I and (B) II.

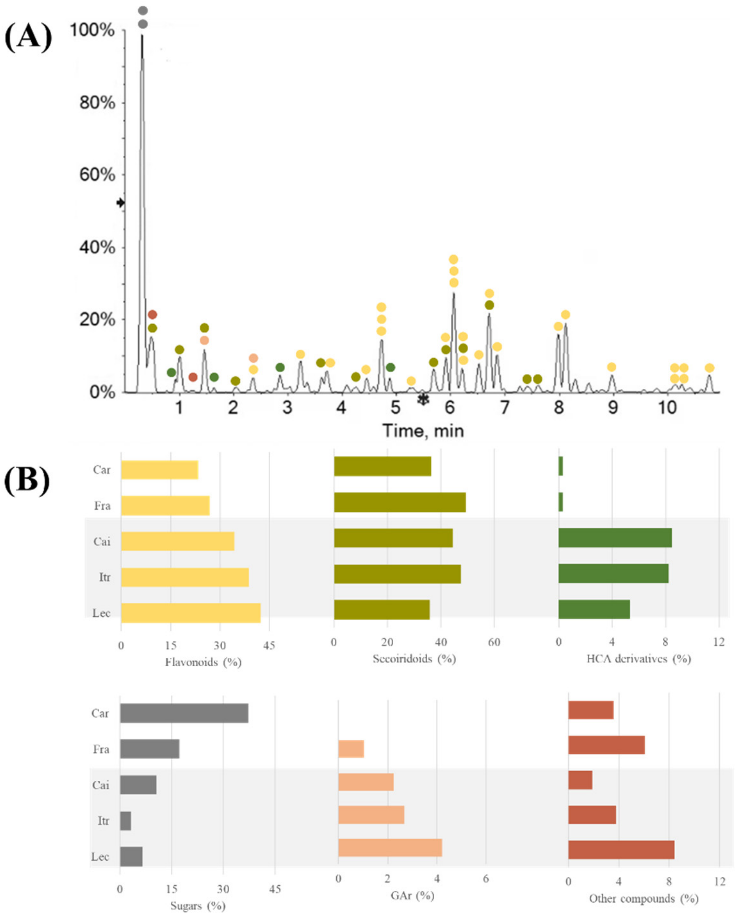

Figure S18. (A) Representative total ion current chromatogram (TIC) of a PLE extract. Compounds are labeled with different colors, based on the class they belong to (the same colors are used for histograms in panel B). (B) Relative abundance (%)of the six metabolite classes under study in each cultivar (Cai = ‘Caiazzana’, Car = ‘Carolea’, Itr = ‘Itrana’, Lec = ‘Leccino’, Fra = ‘Frantoio’).

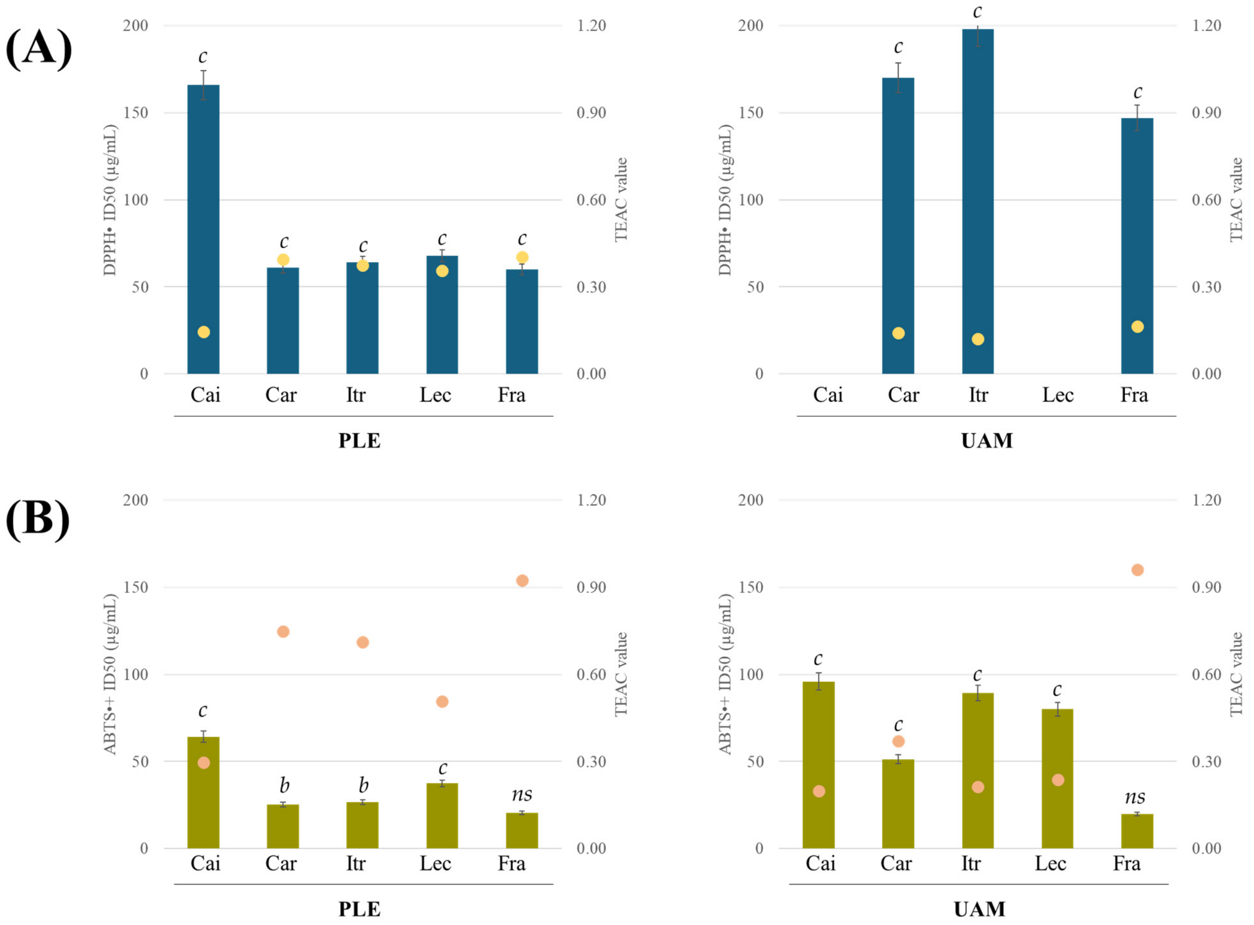

Figure S19. (A) Radical Scavenging Capacity (ID50 and TEAC values) towards (A)DPPH• and (B) ABTS•+ of PLE and UAM extracts from the five olive cultivars under study (Cai = 'Caiazzana', Car = 'Carolea', Itr = 'Itrana', Lec = 'Leccino', Fra = 'Frantoio'). ID50 higher than 200 µg/mL is not reported. Significant differences from the positive control (Trolox®) are highlighted by letters (*a*: $p < 0.05$; *b*: $p < 0.01$; *c*: $p < 0.001$; *ns*: not significant), calculated by GraphPad Prism 8 software (Graphpad Software. La Jolla. CA. USA).

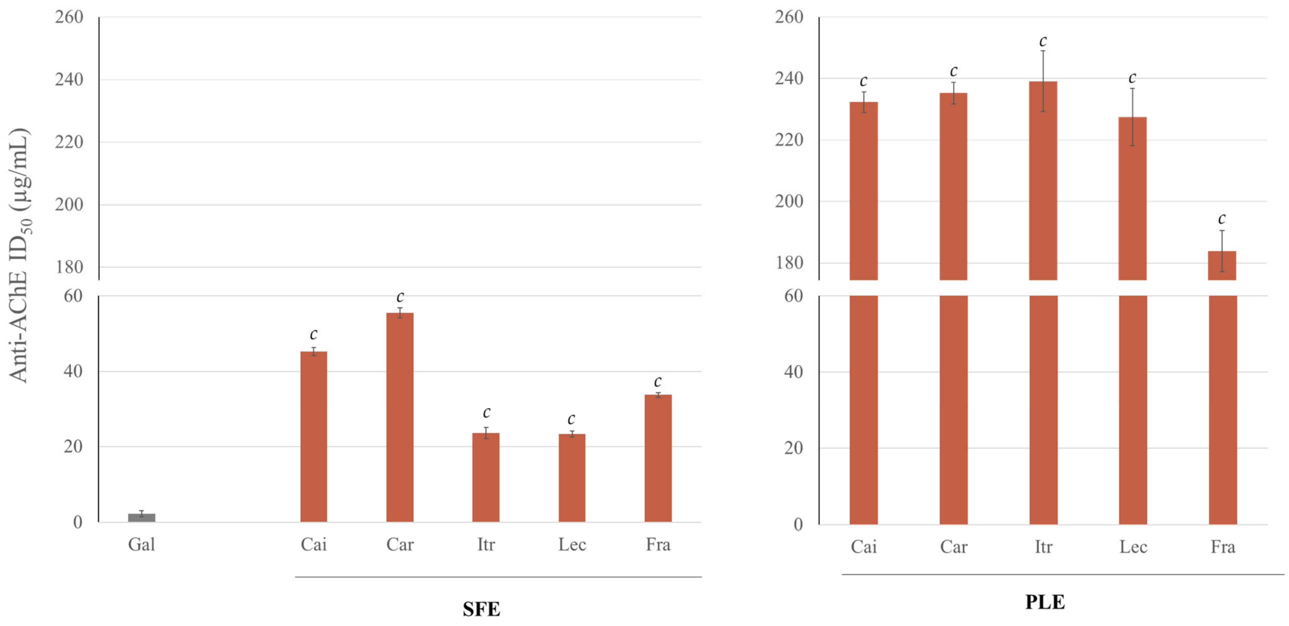

Figure S20. Anti-acetylcholinesterase activity of PLE and SFE extracts from the five olive cultivars under study (Cai = 'Caiazzana', Car = 'Carolea', Itr = 'Itrana', Lec = 'Leccino', Fra = 'Frantoio'). Galantamine (Gal) was used as the positive standard. Significant differences from Gal are highlighted by letters (*a*: $p < 0.05$; *b*: $p < 0.01$; *c*: $p < 0.001$; *ns*: not significant), calculated by GraphPad Prism 8 software (Graphpad Software. La Jolla. CA. USA).
